# Molecular screening of *Entamoeba* spp. (*E. histolytica, E. dispar, E. coli*, and *E. hartmanni*) and *Giardia* intestinalis using PCR and sequencing

**DOI:** 10.1016/j.mex.2023.102361

**Published:** 2023-09-09

**Authors:** M. Tokoro, T. Mizuno, X. Bi, S.A. Lacante, C. Jiang, R.N. Makunja

**Affiliations:** Department of Global Infectious Diseases, Graduate School of Medical Sciences, Kanazawa University, Japan

**Keywords:** Gut protozoan flora, *Entamoeba*, *Giardia*, Molecular epidemiology, PCR-sequencing, PCR-sequencing screening methods for *Entameoba* spp. and *Giardia intestinalis*

## Abstract

A wide range of intestinal protozoan parasites inhabit the human gut. To establish a more comprehensive molecular screening, we designed PCR-sequencing screening methods for *Entamoeba* spp., including commensal species, and *Giardia intestinalis*, and performed such methods using 174 stool samples collected from Kenyan children. The prevalences of the target species were as follows: *E. histolytica* (2/174, 1.1%), *E. dispar* (20/174, 11.5%), *E. coli* (107/174, 61.5%), *E. hartmanni* (77/174, 44.3%), and *G. intestinalis* (54/174, 31.0%). PCR amplicons specific to *G. intestinalis* was differentiated to assemblages A (8/174, 4.6%) and B (46/174, 26.4%). PCR specificity for *Entamoeba* spp. was quite high, except for some cross-reactions between *E. hartmanni* detection primers and *G. intestinalis,* although the false-positive amplicons were discernible by the band size. The 18S rRNA PCR primers that was designed by Monis et al. in 1999 for *G. intestinalis*, have specificity issue, therefore amplicon sequencing was essential not only to determine assemblage classifications but also to confirm the positive results by eliminating potential non-specific reactions. The detection sensitivity of both the *Entamoeba* universal PCR and the *G. intestinalis* PCR was more than 100 copies of the target loci, which is sufficient for detecting a single trophozoite or cyst of both species.

## Graphical abstract

**Figure:**
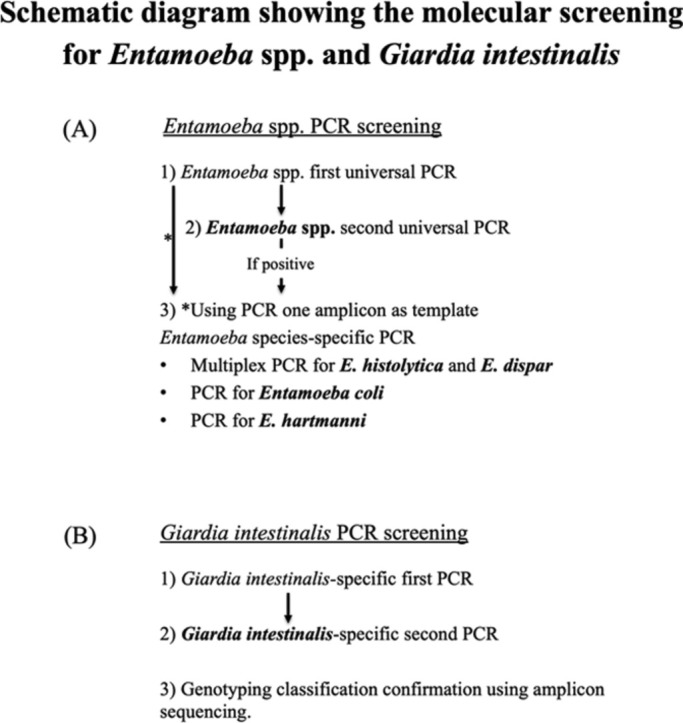

Specifications table

**Table:** 

Subject Area:	Immunology and Microbiology
More specific subject area:	*Parasitology*
Protocol name:	PCR-sequencing screening methods for *Entameoba* spp. and *Giardia intestinalis*
Reagents/tools:	DNAzol^Ⓡ^ reagent (Molecular Research Center, Inc., Cincinnati, OH, USA), proteinase K (Wako Pure Chemical Industries, Osaka, Japan), TaKaRa LA Taq^Ⓡ^ (TaKaRa Bio Inc., Shiga, Japan), TaKaRa LA Taq ^Ⓡ^ with GC Buffer (TaKaRa Bio Inc., Shiga, Japan), 2720 Thermal Cycler (Thermo Fisher Scientific Inc., MA, USA), Agarose S (Nippon Gene, Toyama, Japan), Gel DocTM EZ Imaging System (Bio-Rad Laboratories, Tokyo, Japan), FastGene^Ⓡ^ Gel/PCR Extraction Kit (Nippon Genetics, Tokyo, Japan), BigDye™ Terminator v3.1 Cycle Sequencing Kit (Thermo Fisher Scientific Inc., MA, USA), 3130 Genetic Analyzer (Thermo Fisher Scientific Inc., MA, USA)
Experimental design:	Combination of universal PCRs and subsequent species-specific PCRs with DNA sequencing
Trial registration:	N/A
Ethics:	This study was approved by the scientific and ethics review unit at Kenya Medical Research Institute, Nairobi, Kenya (KEMRI/RES/7/3/1) and Kanazawa University, Japan (2379-1). Informed consent and assent were obtained from the study participants (children) and/or their parents/guardians.
Value of the Protocol:	Detectable all *Entamoeba* species from humans Detectable all assemblages of *G. intestinalis* Comparatively easy and simple sample processing protocol

## Background

In this molecular screening of intestinal protozoan parasites, we initially targeted three main pathogens: *Cryptosporidium* spp., *Entamoeba histolytica*, and *Giardia intestinalis*. However, we did not detect any *Cryptosporidium* (data not shown). Using self-designed universal primer sets for *Entamoeba* screening, we found the presences of various non-pathogenic *Entamoeba* spp., with only two positive results for *E. histolytica*, while the detection rate of *G. intestinalis* was relatively high. The universal polymerase chain reaction (PCR) for *Entamoeba* can detect various *Entamoeba* spp. theoretically, including *E. histolytica, E. coli, E. hartmanni, E. moshkovskii, E. bangladeshi, E. polecki, E. muris, E. nuttalli*, and *E. invadens*. The ability of this method to detect almost all species of *Entamoeba* is an advantage over previous screening methods. In developing tropical areas, a wide range of intestinal protozoan parasites inhabit the human gut. The combination of this universal PCR for *Entamoeba* spp. and the conventional *Giardia* screening PCR could be a convenient tool for molecular screening such commensal species and mild pathogens.

## Experimental design

In this study, we present a comprehensive molecular screening for *Entamoeba* spp. [1] and *G. intestinalis* [2] using stool samples collected from Kenyan children during a molecular epidemiologic investigation.

**Fig. 1:**
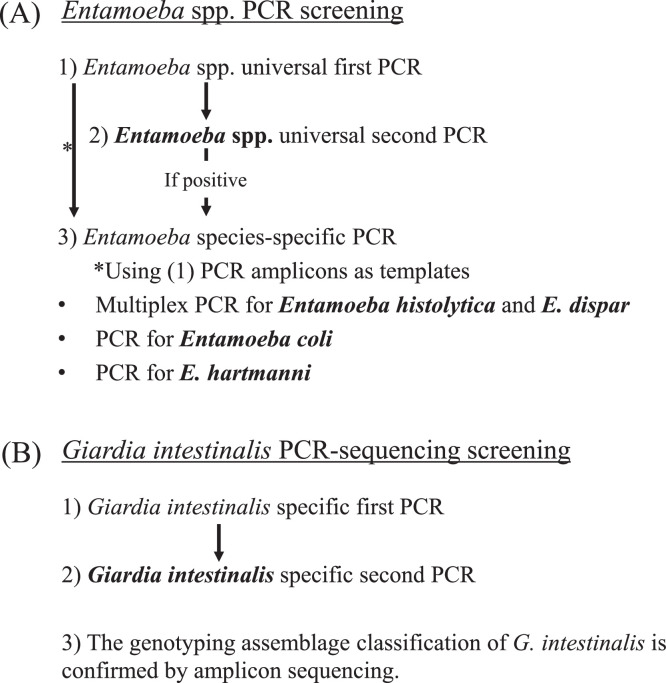
Schematic diagram showing the molecular screening for *Entamoeba* spp. and *Giardia intestinalis*.

(1) Stool sample collection and DNA extraction
  Stool samples were collected from 174 schoolchildren in Kibera, Nairobi, Kenya in November 2017. Briefly, 0.2 g of stool sample was transferred into 1.5 mL screw-capped tubes containing 0.6 mL of DNAzol^Ⓡ^ reagent (Molecular Research Center, Inc., Cincinnati, OH, USA). The collected samples were stored at 20 to 30 °C at the field site and at 4 °C in the laboratory for further analysis.
  Total DNA was extracted from all DNAzol^Ⓡ^ samples according to the manufacturer's instructions with a few modifications [1]. Briefly, the samples were freeze-thawed twice, digested using proteinase K (final concentration 0.4 mg/mL, Wako Pure Chemical Industries, Osaka, Japan) at 55 °C overnight, and then precipitated using the standard ethanol precipitation protocol of the DNAzol^Ⓡ^ reagent. The final DNA precipitate was resuspended in 80 µL of 10 mM Tris-HCl (pH 8.0) containing 1.0 mM ethylenediaminetetraacetic acid and stored at -20 °C until use. This study was approved by the Scientific and Ethics Review Unit of Kenya Medical Research Institute, Nairobi, Kenya (KEMRI/RES/7/3/1), and Kanazawa University, Japan (2379-1). Informed consent and assent were obtained from the study participants (children) and/or their parents or guardians.
(2) *Entamoeba* spp. PCR screening
  For the molecular screening of *Entamoeba* spp. including *E. histolytica, E. dispar, E. coli*, and *E. hartmanni*, a set of universal nested PCR was performed to evaluate whether any *Entamoeba* spp. was present. The positive samples were further evaluated using *Entamoeba* species-specific PCR (Fig. 1A, Table 1).

**Table 1:** Primers targeting the 18S rRNA gene locus of *Entamoeba* spp.

Primer	Oligo sequence and positions on each reference	PCR product (bp)	Reference accession number
***Entamoeba* spp. first universal PCR**			
Amo-F1 (TN21)	5′-^104^YAAAGATTAAGCCATGCATGTST^125^*-3′	1,881 (*E. histolytica)* 1,836 (*E. dispar)* 2,044 (*E. coli)* 1,898 (*E. hartmanni)*	AB282660 AB282661 AB444953 KX618191
Amo-R1 (TN14)	5′-^1984^AWACCTTGTTACGACTTCTY^1965^*-3′		

***Entamoeba* spp. second universal PCR**			
Amo-F2 (MA115)	5′-^438^GAYWTCGGAGAGGGAGCT^455^*-3′	1,191 (*E. histolytica)* 1,201 (*E. dispar)* 1,310 (*E. coli)* 1,205 (*E. hartmanni)*	AB282660 AB282661 AB444953 KX618191
Amo-R2 (SA12)	5′-^1636^GCGTGCRGCCCAAGATG^1620^*-3′		

***E. histolytica* (Eh)- and *E. dispar* (Ed)-specific multiplex PCR**			
Eh-L	5′-^714^ACATTTTGAAGACTTTATGTAAGTA^738^-3′	427	AB282660
Eh-R	5′-^1140^CAGATCTAGAAACAATGCTTCTCT^1117^-3′		
Ed-L	5′-^1534^GTTAGTTATCTAATTTCGATTAGAA^1558^-3′	194	AB282661
Ed-R	5′-^1727^ACACCACTTACTATCCCTACC^1708^-3′		

***E. coli* (Ec)-specific PCR**			
Ec-F (MA113)	5′-^289^GCCAAGAGAATTGTAGAAATCG^310^-3′	349	AB444953
Amo-R3 (TA28)	5′-^637^CACTATTGGAGCTGGAATTAC^618^-3′		

***E. hartmanni* (Er)*-*specific PCR**			
Er-F (MA67)	5′-^147^TTGGATGTAGAGATACATTC^166^-3′	419	KX618191
Amo-R3 (TA28)	5′-^565^CACTATTGGAGCTGGAATTAC^546^-3′		

⁎ Position numbers are based on the *E. histolytica* reference sequence (AB282660).
  For the universal *Entamoeba* spp. screening, a region of the 18S rRNA gene was amplified using nested PCR with the primers Amo-F1(TN21) and Amo-R1(TN14) in the first round and Amo-F2(MA115) and Amo-R2 (SA12) in the second round (Table 1) [1]. It is worth mentioning that these universal primers were also 100% matched to the target genomic DNA region of other *Entamoeba* species, such as *E. moshkovskii* (KP722601), *E. bangladeshi* (KR025412), *E. polecki* (LC230018), *E. muris* (AB445018), *E. nuttalli* (AB282657), and *E. invadens* (KR025413). PCR amplification was performed in a 10 µl reaction mixture containing 1 × LA Taq^Ⓡ^ PCR buffer, 0.5 U LA Taq^Ⓡ^ polymerase (TaKaRa Bio Inc., Shiga, Japan), approximately 100 ng of the extracted DNA (1 µL) as the template, 0.4 µM of the forward and reverse primers, and 0.5 mM of deoxynucleotide triphosphates (dNTPs) with 0.1% dimethyl sulfoxide. The second round of PCR was performed under the same conditions as the first round PCR, except that 1.0 µL of the amplicon from the first round of PCR was used as the template. The cycling parameters for the first and second rounds of PCR are listed in Table 2.

**Table 2:** PCR conditions.

PCR reaction	Primers	PCR condition
***Entamoeba* spp. first universal PCR**	Amo-F1(TN21), Amo-R1(TN14)	1 cycle at 94 °C for 3 min 30 cycles at 94 °C for 30 s, 58 °C for 30 s, and 72 °C for 3 min 1 cycle at 72 °C for 5 min 1 cycle at 94 °C for 3 min,

***Entamoeba* spp. second universal PCR**	Amo-F2(MA115), Amo-R2 (SA12)	1 cycle at 94 °C for 3 min, 35 cycles at 94 °C for 30 s, 57 °C for 30 s, and 72 °C for 90 s 1 cycle at 72 °C for 5 min

***E. histolytica-* and *E. dispar*-specific multiplex PCR**		
***E. histolytica***	Eh-L, Eh-R	1 cycle at 94 °C for 3 min 35 cycles at 94 °C for 30 s, 57 °C for 30 s, and 72 °C for 30 s 1 cycle at 72 °C for 5 min
***E. dispar***	ED-L, Ed-R	

***E. coli*-specific PCR**	Ec-F(MA113), Amo-R3(TA28)	1 cycle at 94 °C for 3 min 35 cycles at 94 °C for 30 s, 57 °C for 30 s, and 72 °C for 30 s 1 cycle at 72 °C for 5 min

***E. hartmanni-*specific PCR**	Er-F(MA67), Amo-R3(TA28)	1 cycle at 94 °C for 3 min 35 cycles at 94 °C for 30 s, 57 °C for 30 s, and 72 °C for 30 s 1 cycle at 72 °C for 5 min

***Giardia intestinalis*-specific first PCR**	KN63, KN62	1 cycle at 94 °C 3 min 35 cycles at 94 °C for 30 s, 57 °C for 30 s, and 72 °C for 30 s 1 cycle at 72 °C for 5 min

***G. intestinalis*-specific second PCR**	KN63, KN64	1 cycle at 94 °C for 3 min 35 cycles at 94 °C for 30 s, 57 °C for 30 s, and 72 °C for 30 s 1 cycle at 72 °C for 5 min
  For *Entamoeba* species-specific PCR, specific multiplex primer sets (Eh-L, Eh-R, Ed-L, and Ed-R) were used for *E. histolytica* (product size, 427 bp) and *E. dispar* (194 bp), which were designed by Evangelopoulos et al. [3], while all other species-specific primers were self-designed [1]. The species-specific primers used are listed in Table 1. The Amo-R3 primer sequence was 100% matched to the target regions in *E. histolytica* (AB282660), *E. dispar* (AB282661), *E. coli* (AB444953), *E. hartmanni* (KX618191), *E. moshkovskii* (KP722601), *E. bangladeshi* (KR025412), *E. polecki* (LC230018), *E. muris* (AB445018), and *E. nuttalli* (AB282657). This means that the specificities of the PCRs for *E. coli* and *E. hartmanni* were ensured by the primers Ec-F(MA113) and Er-F(MA67), respectively. Same PCR conditions as those used in the second round of universal PCR were performed (Table 2). All PCR cycles were conducted using the 2720 Thermal Cycler (Thermo Fisher Scientific Inc., MA, USA).
  Although all PCR specificities were confirmed in a previous study [1], we observed slightly higher bands than the true positive one on the gel image of *E. hartmanni-*specific PCR in this study. For sequencing analyses, those slightly higher-seized PCR products were excised from the agarose gel after electrophoresis and then purified using the FastGene^Ⓡ^ Gel/PCR Extraction Kit (Nippon Genetics, Tokyo, Japan). The purified amplicons were sequenced using amplification primers and/or appropriate sequencing primers on the 3130 Genetic Analyzer (Thermo Fisher Scientific Inc., MA, USA) using the BigDye^Ⓡ^ Terminator v3.1 Cycle Sequencing Kit (Thermo Fisher Scientific Inc.). All confirmed DNA sequences were registered in a DNA database [DNA Data Bank of Japan (DDBJ), European Molecular Biology Laboratory (EMBL), GenBank], with accession numbers LC732502–LC732508. The slightly larger PCR products amplified using *E. hartmanni*-specific primers were all identified as products resulting from cross-reaction with giardial DNA.
(3) *Giardia intestinalis* PCR screening and sequencing
  The semi-nested and species-specific molecular screening of *Giardia intestinalis* were conducted, and genotyping was confirmed using the DNA sequences of the positive amplicons (Fig. 1B, Table 3).

**Table 3:** Primers targeting the 18S rRNA gene locus of *Giardia intestinalis*.

Primer	Oligo sequence and positions on each reference	PCR product (bp)	Reference accession number
***G. intestinalis*-specific first PCR**			
Gia-F (KN63)	5′-^3^TCCGGTYGATCCTGCCG^19^-3′	464	AF199447*
G18S3 (KN62)	5′-^466^CTGGAATTACCGCGGCTGCT^447^-3′		

***G. intestinalis*-specific second PCR**			
Gia-F (KN63)	5′-^3^TCCGGTYGATCCTGCCG^19^-3′	382	AF199447
Gia-R2 (MA64)	5′-^384^CCCGTCGCTGCCTCGCG^368^-3′		

⁎ AF199447: partial sequence, small subunit ribosomal RNA gene of *G. intestinalis* isolate BAH12c14.
  For the *G. intestinalis*-specific primers, the first PCR primer set was designed based on a set of primers G18S2 (5′-TCCGGTYGATT*CTGCC-3′) and G18S3 (5′-CTGGAATTACCGCGGCTGCT-3′), which was originally designed by Monis et al. [4]. However, the “T*” on the primer did not match the conserved region of the current giardial sequence alignment and an additional “G” was further conserved at the 3′-end. Therefore, in this study, we modified the G18S2 primer to Gia-F(KN63) (5′- TCCGGTYGATCCTGCCG-3′). For the second round of *G. intestinalis*-specific PCR, the inner region of the first PCR product was targeted, and the primer set Gia-F(KN63) (5′-TCCGGTYGATCCTGCCG-3′) and Gia-R2 (MA64) (5′-CCCGTCGCTGCCTCGCG-3′) was designed and used.
  PCR screening was performed in a 10 µl reaction mixture containing 1 × GC buffer I, 0.4 µM of each primer, 0.5 mM of each deoxynucleotide triphosphate (dNTP), and 0.5 U of TaKaRa LA Taq ^Ⓡ^ with GC Buffer (TaKaRa Bio Inc.), with 5% dimethyl sulfoxide as an additive. The cycling parameters for the first and second PCRs are listed in Table 2.
  To confirm the identity of *G. intestinalis* and the assemblage classification of *G. intestinalis*, all DNA sequences of the second PCR amplicons were determined directly using Gia-F (KN63) and/or Gia-R2 (MA64) primers. Purification and DNA sequencing methods were the same as those described above for *Entamoeba* species. For the assemblage classification of *G. intestinalis*, the alignment of amplicon sequences and Bayesian Inference analyses were conducted using Geneious 10.2.3 (Biomatters Ltd., Auckland, New Zealand). All confirmed 18S rRNA partial sequences of *G. intestinalis* were registered in the DNA database (DDBJ/EMBL/GenBank) with accession numbers LC732448–LC732501.
(4) PCR sensitivity test for the universal PCR targeting 18S rRNA gene locus for *Entamoeba* spp. and *G. intestinalis*
  To conduct sensitivity tests, we constructed recombinant plasmids of pMD20-T using amplicons from the first PCR targeting the 18S rRNA gene locus. Specifically, we used a 1881-bp amplicon by primers Amo-F1(TN21) and Amo-R1(TN14) to test for *E. histolytica*, and a 464-bp amplicon by primers Gia-F (KN63) and G18S3 (KN62) to test for *G. intestinalis*. The TaKaRa TA-Mighty Cloning Kit (Takara Bio Inc.) was used for cloning following the manufacturer's instructions. The resulting recombinant plasmids (pMD20-T with those amplicons mentioned above) were transformed into *Escherichia coli* DH5-α and screened on Luria Broth (LB) agar plates containing 100 mg/L Ampicillin. Positive colonies were cultured overnight in LB liquid medium with 100 mg/L Ampicillin and then purified using the QIAGEN^Ⓡ^ plasmid mini kit (Qiagen K.K., Tokyo, Japan) according to the manufacturer's protocol. The full-length insertion DNA was sequenced using M13 primer RV and M13 primer M4, and the amplicons were confirmed. The concentration of the plasmids was measured as double-stranded (ds) DNA using the OD 260 nm of µDrop™ (Thermo Fisher Scientific K.K., Tokyo, Japan), and the copy numbers were calculated based on their molecular weights. The detection limits of the universal PCR screenings for *Entamoeba* spp. and *G. intestinalis* were evaluated using dilution series of the templates.

## Reagents and tools

DNAzol^Ⓡ^ reagent (Molecular Research Center, Inc., Cincinnati, OH, USA)

Wako Pure Chemical Industries, Osaka, Japan)

TaKaRa LA Taq^Ⓡ^ (TaKaRa Bio Inc., Shiga, Japan)

TaKaRa LA Taq ^Ⓡ^ with GC Buffer (TaKaRa Bio Inc., Shiga, Japan)

TaKaRa TA-Mighty Cloning Kit (TaKaRa Bio Inc., Shiga, Japan)

2720 Thermal Cycler (Thermo Fisher Scientific Inc., MA, USA)

Agarose S (Nippon Gene, Toyama, Japan)

Gel DocTM EZ Imaging System (Bio-Rad Laboratories, Tokyo, Japan)

FastGene^Ⓡ^ Gel/PCR Extraction Kit (Nippon Genetics, Tokyo, Japan)

BigDye™ Terminator v3.1 Cycle Sequencing Kit (Thermo Fisher Scientific Inc., MA, USA)

3130 Genetic Analyzer (Thermo Fisher Scientific Inc., MA, USA)

µDrop™ (Thermo Fisher Scientific K.K., Tokyo, Japan)

Geneious 10.2.3 (Biomatters Ltd., Auckland, New Zealand)

## Data measurement and analysis

**Fig. 2:**
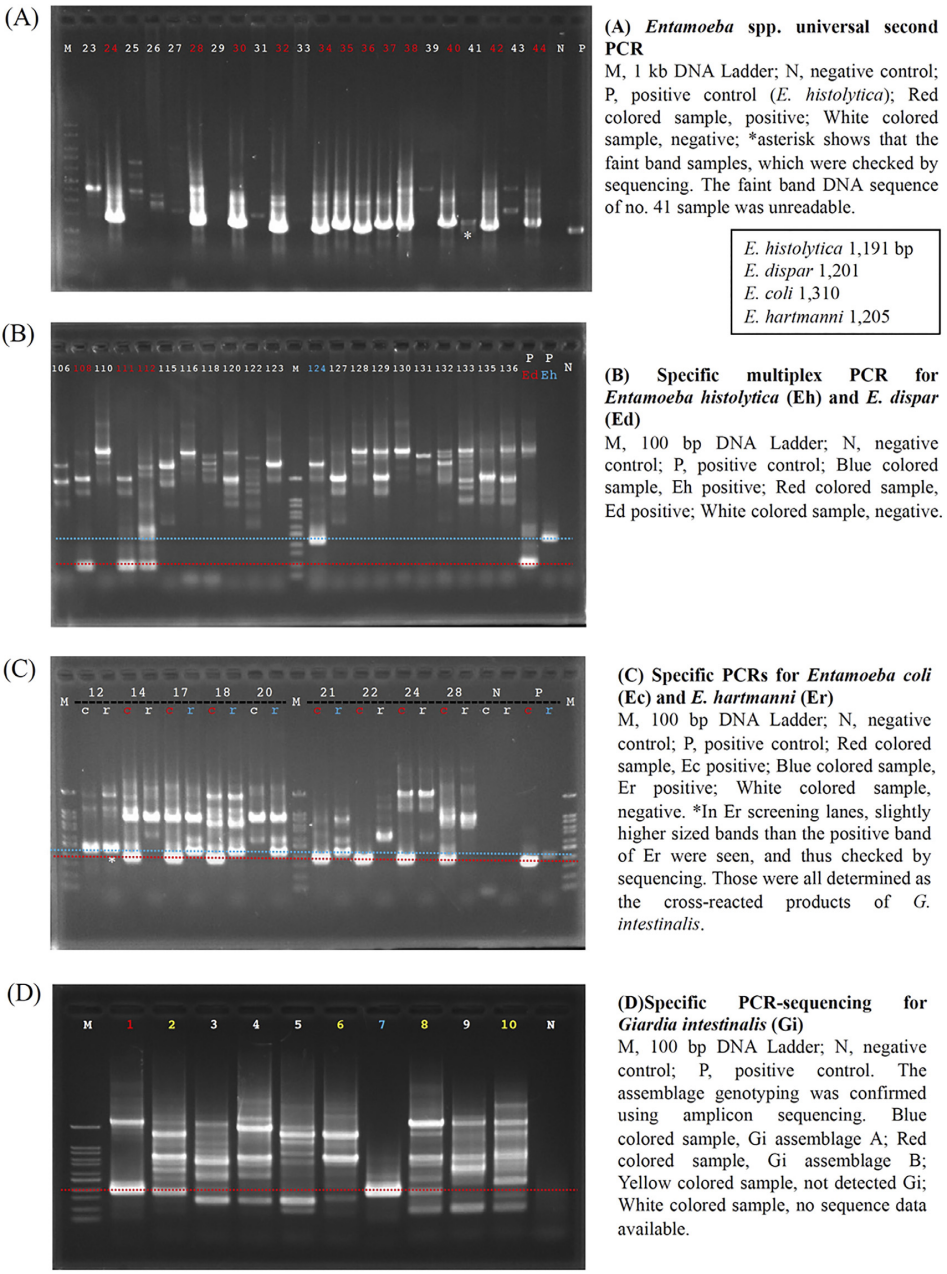
Representative images of PCR screening gel-electrophoresis.

PCR products of the universal PCR screening for *Entamoeba* spp. were electrophoresed on 2.0% agarose S gels (Nippon Gene, Toyama, Japan), though other PCR products were all electrophoresed on 1.0% agarose gels and visualized using ethidium bromide on a Gel Doc^TM^ EZ Imaging System (Bio-Rad Laboratories, Tokyo, Japan). All electrophoresed images are shown in the Supplementary Figs. 1–4, and only representative images are shown in Fig. 2).

**(A) *Entamoeba* spp. second universal PCR**. M, 1 kb DNA Ladder; N, negative control; P, positive control (*E. histolytica*: 1,191bp); red colored sample, positive; white colored sample, negative; *asterisk shows that the faint band samples, which were verified via sequencing. The faint band DNA sequence of no. 41 sample was unreadable. **(B) Specific multiplex PCR for *Entamoeba histolytica* (Eh) and *E. dispar* (Ed)**. M, 100 bp DNA Ladder; N, negative control; P, positive control; blue colored sample, Eh positive: 427 bp; red colored sample, Ed positive: 194 bp; white colored sample, negative. **(C) Specific PCRs for *Entamoeba coli* (Ec) and *E. hartmanni* (Er)**. M, 100 bp DNA Ladder; N, negative control; P, positive control; Red colored sample, Ec positive: 349 bp; Blue colored sample, Er positive: 419 bp; white colored sample, negative. *In Er screening lanes, slightly higher sized bands than the positive band of Er were observed, and thus checked via sequencing. Those were all determined as the cross-reacted products of *G. intestinalis* (sample 12: Gi cross-reaction LC732502). **(D) Specific PCR for *Giardia intestinalis* (Gi) and sequencing for assemblages A and B**. M, 100 bp DNA Ladder; N, negative control; P, positive control: 382 bp. The assemblage genotyping was confirmed using amplicon sequencing. Blue colored sample, Gi assemblage A; red colored sample, Gi assemblage B; yellow colored sample, sequenced but not detected Gi; white colored sample, no sequence data available.

**Fig. 3:**
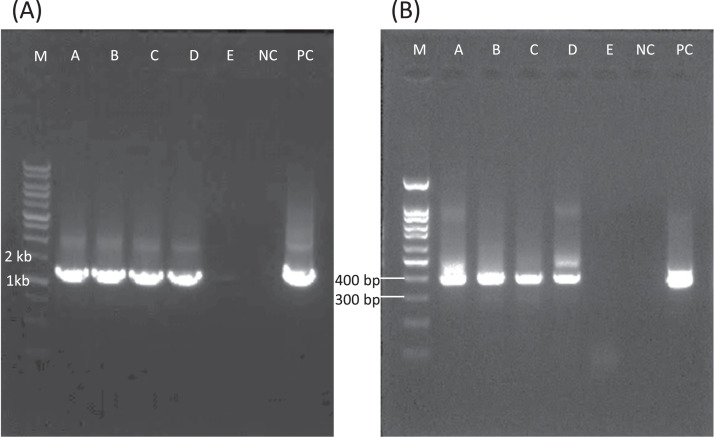
The results of PCR sensitivity test. (A) The second PCR results of universal PCR targeting 18S rRNA gene locus for *Entamoeba* spp. [*E. histolytica* (1,191 bp)]. M: Marker (1-kb DNA ladder); Copy number of a test locus A: 1.0 × 10^5^, B: 1.0 × 10^4^, C: 1.0 × 10^3^, D: 1.0 × 10^2^, E: 10; NC: negative control; PC: positive control. (B) The second PCR results of universal PCR targeting 18S rRNA gene locus for *G. intestinalis* (382 bp). M: Marker (100-bp DNA ladder); Copy number of a test locus A: 1.0 × 10^5,^ B: 1.0 × 10^4^, C: 1.0 × 10^3^, D: 1.0 × 10^2^, E: 10; NC: negative control; PC: positive control.

(1) *Entamoeba* spp. second universal PCR (Fig. 2A)
  The positive bands of *Entamoeba* spp. varied in size: *E. histolytica* (1,192 bp), *E. dispar* (1,201 bp), *E. hartmanni* (1,205 bp), and *E. coli* (1,310 bp). The band intensity was generally strong; therefore, positive identification was relatively clear. Faint bands that rarely appeared were considered as non-specific reaction results because sequencing did not identify any cases of *Entamoeba* species.
(2) Multiplex PCR specific for *E. histolytica* and *E. dispar* (Fig. 2B)
  Gel imaging revealed various non-specific amplicon bands; however, the size of non-specific bands was generally more than 500 bp, and thus the positive bands of *E. histolytica* (427 bp) and *E. dispar* (194 bp) were distinguishable. In previous sequencing assessments [1], no cases of cross-reactions were observed in those amplicon bands derived from other organisms (data not shown).
(3) *E. coli-* and *E. hartmanni-*specific PCR (Fig. 2C)
  Gel imaging revealed various non-specific amplicon bands; however, the size of such cross-reacted bands was more than 500 bp. Although problematic cross-reacted bands were not observed for the *E. coli* positive band (349 bp), slightly higher-sized bands than the positive band (419 bp) of *E. hartmanni* appeared. These higher-sized bands were all identified as cross-reacted products with *G. intestinalis*. For example, the band of sample 12, which was identified as the cross-reacted partial 18S rRNA gene fragment of *G. intestinalis* (LC732502). All other images of these cross reactions are shown in Supplementary Fig. 2. Even slightly, the band size was different from that of the true positive one; thus, this cross-reaction was distinguishable from the positive control.
(4) Specific PCR for *Giardia intestinalis* and sequencing for assemblages A and B (Fig. 2D)
  Various non-specific amplicon bands were observed at the position of true positive band of *G. intestinalis* during gel imaging. To confirm the presence of *Giardia*, all cross-reacted samples should be eliminated by sequence evaluation. For example, sample 1 was the assemblage B of *G. intestinalis*, sample 7 was assemblage A of *G. intestinalis*, and samples 2, 6, 8, and 10 were unreadable by sequencing. Non-specific band amplification seemed to be suppressed by the positive band amplification of the true *Giardia* template, probably due to the competitive use of nucleotide materials; however, sequencing is required to confirm the result. Assemblages of *G. intestinalis* were determined using those *G. intestinalis* positive amplicon reads by the alignment analysis (Supplementary Fig. 5).
(5) The sensitivity of the universal screening PCRs targeting the 18S rRNA gene locus for *Entamoeba* spp. and *G. intestinalis*
  Universal PCR screening for *E. histolytica* and *G. intestinalis* were both able to detect more than 100 copies of tested locus of DNA templates (Fig. 3).

The 18S rRNA gene locus of *E. histolytica* is known to contain at least 200 copies in the cytoplasm as a plasmid [5], and therefore, this screening PCR might retain sufficient sensitivity to detect a single trophozoite or cyst of *E. histolytica*.

*G. intestinalis* possesses five major chromosomes that are capped at both ends by telomeric tandem repeats containing 18S rRNA gene locus [6]. Even with a minimum of two repeats, each genome of *G. intestinalis* could possess at least 20 copies of the 18S rRNA locus. Additionally, due to the tetraploid nature of giardial karyotype, each trophozoite maintains at least 80 copies of the 18S rRNA locus. Considering these factors, the screening PCR's sensitivity might be sufficient for detecting a single trophozoite or cyst of *G. intestinalis*.

## Conclusion

A comprehensive molecular screening method was performed using 174 stool samples collected from Kenyan children. The prevalence of *Entamoeba* spp. and *G. intestinalis* were as follows: *E. histolytica* (2/174, 1.1%), *E. dispar* (20/174, 11.5%), *E. coli* (107/174, 61.5%), *E. hartmanni* (77/174, 44.3%), and *G. intestinalis* (54/174, 31.0%). Sequencing of PCR amplicons specific to *G. intestinalis* (382 bp) could differentially identified assemblages A (8/174, 4.6%) and B (46/174, 26.4%). The specificity of all PCR cycles for *Entamoeba* spp. was quite high, except for some cross-reactions of *E. hartmanni* detection primers with *G. intestinalis,* although the false-positive amplicons were discernible by the band size. In *G. intestinalis* screening, amplicon sequencing is generally required not only to determine assemblage classifications but also to confirm positive results by eliminating potential non-specific reactions. The detection sensitivity of both the *Entamoeba* universal PCR and the *G. intestinalis* PCR was more than 100 copies of the target loci, which is sufficient for detecting a single trophozoite or cyst of both species.

## Funding

This study was supported by 10.13039/501100007807Japan Society for the promotion of Science [JSPS 16H05842 (XB)] and, in part, by the 10.13039/501100005993Grant-in-Aid for Scientific Research [25460514 (MT)] from the Ministry of Education, Culture, Sports, Science and Technology of Japan. This project was also partially supported by the Research Program on Emerging and Re-emerging Infectious Diseases from Japan 10.13039/100009619Agency for Medical Research and Development, AMED [22fk0108639s0301 (MT)].

## CRediT authorship contribution statement

**M. Tokoro:** Project administration, Conceptualization, Writing – original draft, Writing – review & editing. **T. Mizuno:** Investigation, Data curation, Formal analysis, Validation, Supervision. **X. Bi:** Funding acquisition, Data curation, Formal analysis, Validation. **S.A. Lacante:** Methodology, Visualization, Investigation. **C. Jiang:** Methodology, Investigation, Data curation. **R.N. Makunja:** Methodology, Investigation, Resources.

## Declaration of Competing Interest

The authors declare that they have no known competing financial interests or personal relationships that could have influenced the work reported in this study.

## Data Availability

Data will be made available on request.

## Appendix Supplementary materials

Image, application 1
