# Supplementary material for: Molecular screening of Entamoeba spp. (E. histolytica, E. dispar, E. coli, and E. hartmanni) and Giardia intestinalis using PCR and sequencing
Source: MethodsX. 2023 Sep 9;11:102361. doi: 10.1016/j.mex.2023.102361 (PMC10511480; doi:10.1016/j.mex.2023.102361)
Supplement: Supplementary file 1 [file mmc1.pptx]

## Slide 1
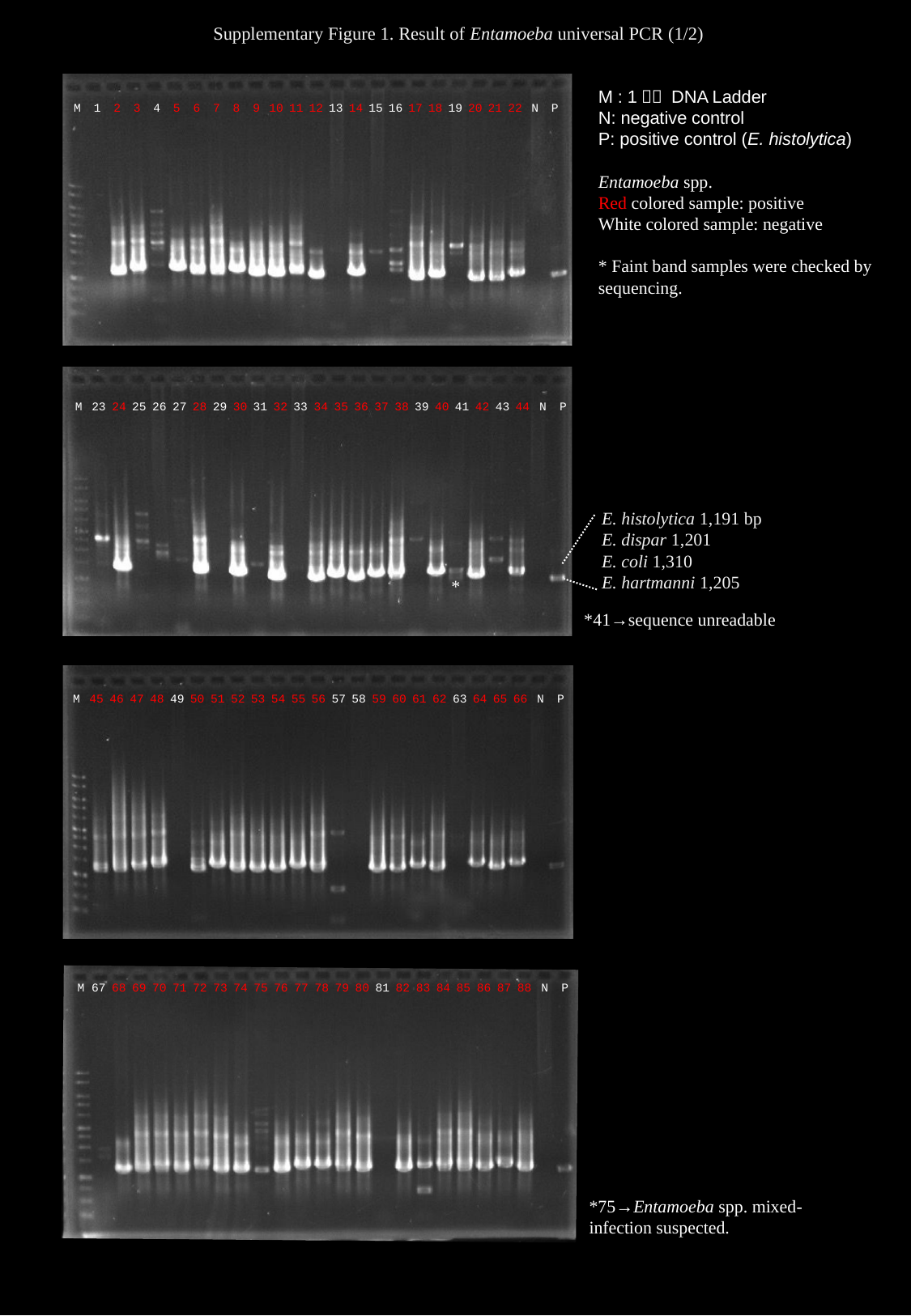

# Supplementary Figure 1. Result of Entamoeba universal PCR (1/2)
M : 1ｋｂ DNA Ladder
N: negative control
P: positive control (E. histolytica)
Entamoeba spp.
Red colored sample: positive
White colored sample: negative
* Faint band samples were checked by sequencing.
| M | 1 | 2 | 3 | 4 | 5 | 6 | 7 | 8 | 9 | 10 | 11 | 12 | 13 | 14 | 15 | 16 | 17 | 18 | 19 | 20 | 21 | 22 | N | P |
| --- | --- | --- | --- | --- | --- | --- | --- | --- | --- | --- | --- | --- | --- | --- | --- | --- | --- | --- | --- | --- | --- | --- | --- | --- |
| M | 23 | 24 | 25 | 26 | 27 | 28 | 29 | 30 | 31 | 32 | 33 | 34 | 35 | 36 | 37 | 38 | 39 | 40 | 41 | 42 | 43 | 44 | N | P |
| --- | --- | --- | --- | --- | --- | --- | --- | --- | --- | --- | --- | --- | --- | --- | --- | --- | --- | --- | --- | --- | --- | --- | --- | --- |
E. histolytica 1,191 bp
E. dispar 1,201
E. coli 1,310
E. hartmanni 1,205
*
*41→sequence unreadable
| M | 45 | 46 | 47 | 48 | 49 | 50 | 51 | 52 | 53 | 54 | 55 | 56 | 57 | 58 | 59 | 60 | 61 | 62 | 63 | 64 | 65 | 66 | N | P |
| --- | --- | --- | --- | --- | --- | --- | --- | --- | --- | --- | --- | --- | --- | --- | --- | --- | --- | --- | --- | --- | --- | --- | --- | --- |
| M | 67 | 68 | 69 | 70 | 71 | 72 | 73 | 74 | 75 | 76 | 77 | 78 | 79 | 80 | 81 | 82 | 83 | 84 | 85 | 86 | 87 | 88 | N | P |
| --- | --- | --- | --- | --- | --- | --- | --- | --- | --- | --- | --- | --- | --- | --- | --- | --- | --- | --- | --- | --- | --- | --- | --- | --- |
*75→Entamoeba spp. mixed-infection suspected.

## Slide 2
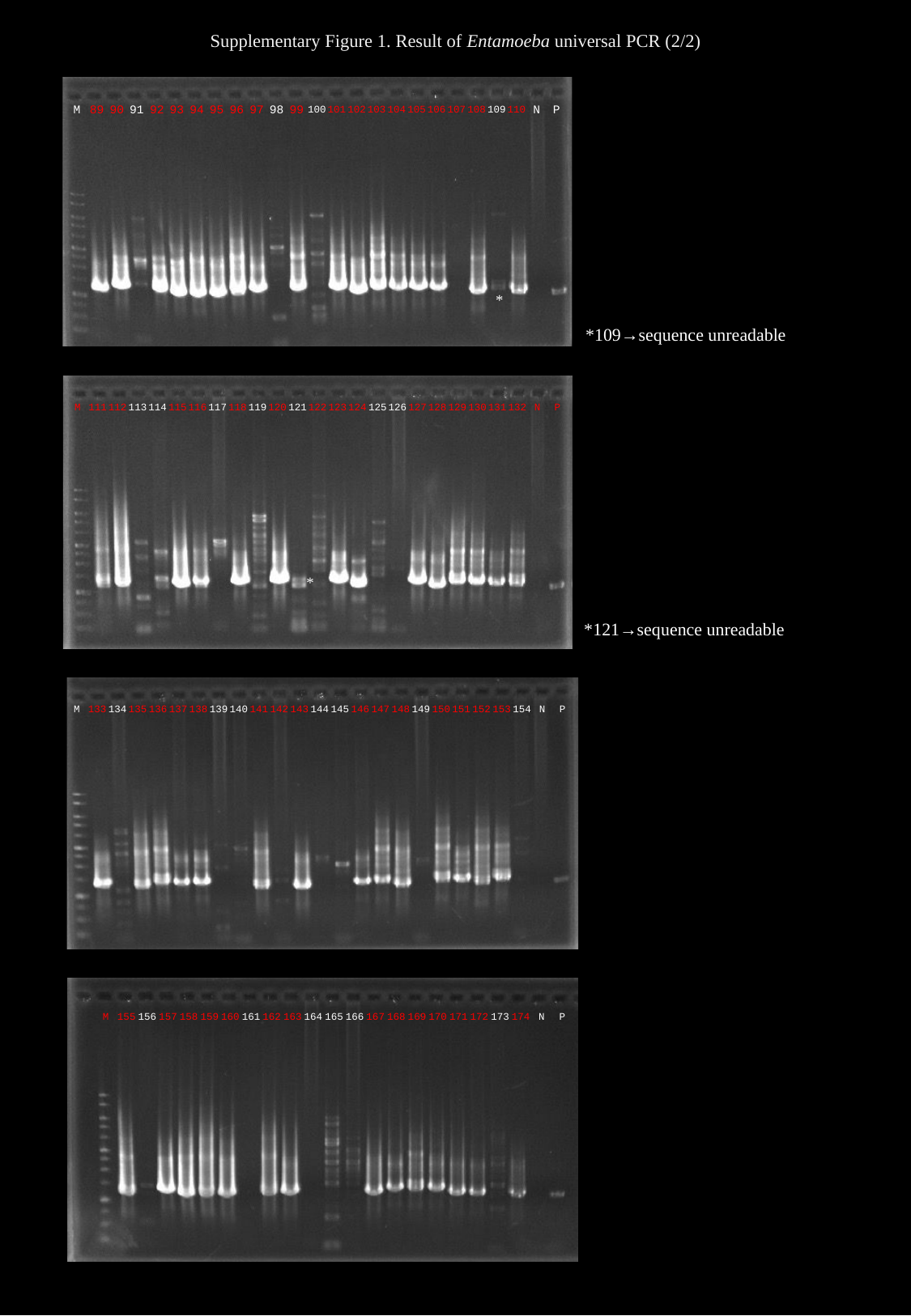

Supplementary Figure 1. Result of Entamoeba universal PCR (2/2)
| M | 89 | 90 | 91 | 92 | 93 | 94 | 95 | 96 | 97 | 98 | 99 | 100 | 101 | 102 | 103 | 104 | 105 | 106 | 107 | 108 | 109 | 110 | N | P |
| --- | --- | --- | --- | --- | --- | --- | --- | --- | --- | --- | --- | --- | --- | --- | --- | --- | --- | --- | --- | --- | --- | --- | --- | --- |
*
*109→sequence unreadable
| M | 111 | 112 | 113 | 114 | 115 | 116 | 117 | 118 | 119 | 120 | 121 | 122 | 123 | 124 | 125 | 126 | 127 | 128 | 129 | 130 | 131 | 132 | N | P |
| --- | --- | --- | --- | --- | --- | --- | --- | --- | --- | --- | --- | --- | --- | --- | --- | --- | --- | --- | --- | --- | --- | --- | --- | --- |
*
*121→sequence unreadable
| M | 133 | 134 | 135 | 136 | 137 | 138 | 139 | 140 | 141 | 142 | 143 | 144 | 145 | 146 | 147 | 148 | 149 | 150 | 151 | 152 | 153 | 154 | N | P |
| --- | --- | --- | --- | --- | --- | --- | --- | --- | --- | --- | --- | --- | --- | --- | --- | --- | --- | --- | --- | --- | --- | --- | --- | --- |
| M | 155 | 156 | 157 | 158 | 159 | 160 | 161 | 162 | 163 | 164 | 165 | 166 | 167 | 168 | 169 | 170 | 171 | 172 | 173 | 174 | N | P |
| --- | --- | --- | --- | --- | --- | --- | --- | --- | --- | --- | --- | --- | --- | --- | --- | --- | --- | --- | --- | --- | --- | --- |

## Slide 3
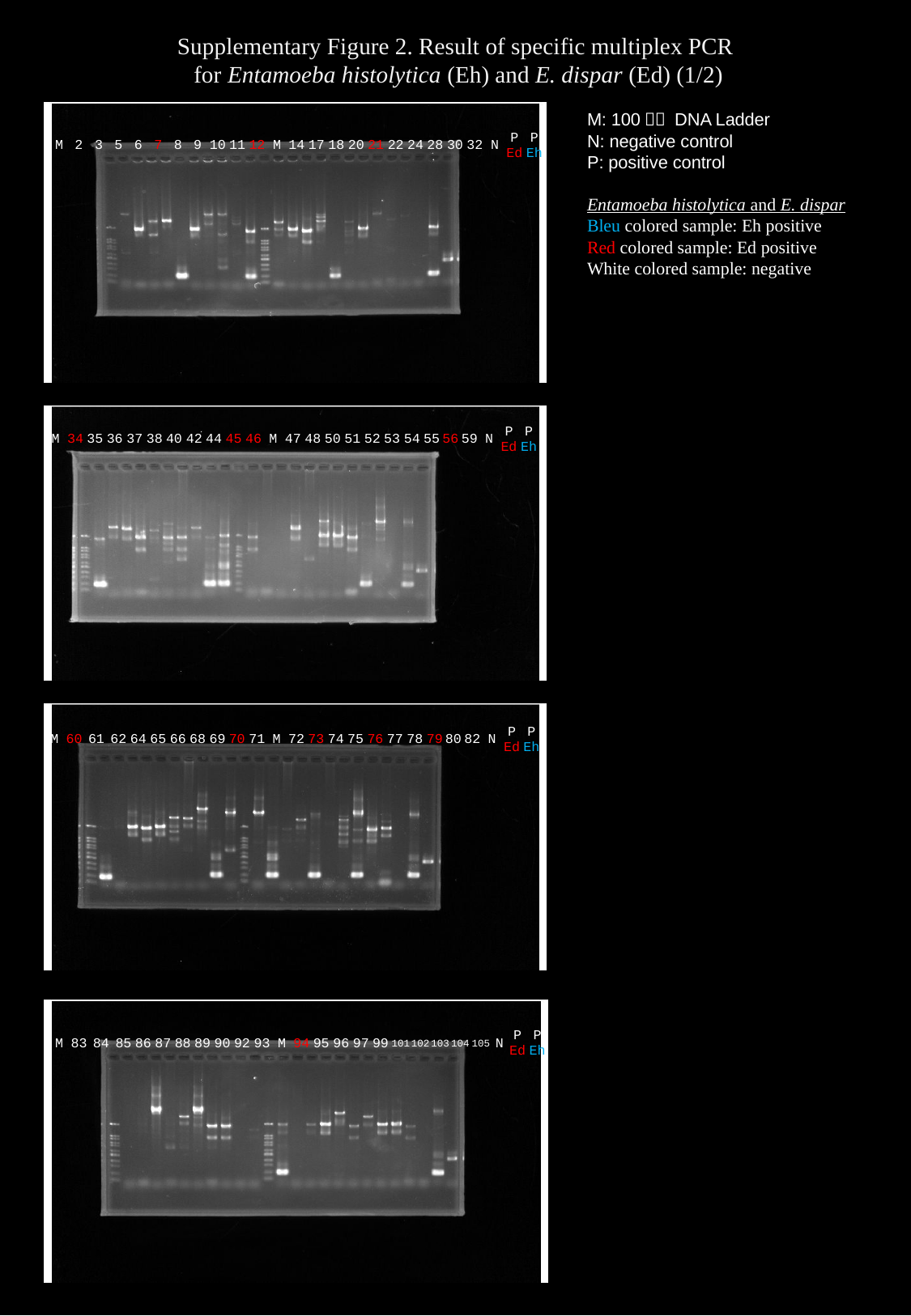

Supplementary Figure 2. Result of specific multiplex PCR
for Entamoeba histolytica (Eh) and E. dispar (Ed) (1/2)
M: 100ｂｐ DNA Ladder
N: negative control
P: positive control
Entamoeba histolytica and E. dispar
Bleu colored sample: Eh positive
Red colored sample: Ed positive
White colored sample: negative
| M | 2 | 3 | 5 | 6 | 7 | 8 | 9 | 10 | 11 | 12 | M | 14 | 17 | 18 | 20 | 21 | 22 | 24 | 28 | 30 | 32 | N | P Ed | P Eh |
| --- | --- | --- | --- | --- | --- | --- | --- | --- | --- | --- | --- | --- | --- | --- | --- | --- | --- | --- | --- | --- | --- | --- | --- | --- |
| M | 34 | 35 | 36 | 37 | 38 | 40 | 42 | 44 | 45 | 46 | M | 47 | 48 | 50 | 51 | 52 | 53 | 54 | 55 | 56 | 59 | N | P Ed | P Eh |
| --- | --- | --- | --- | --- | --- | --- | --- | --- | --- | --- | --- | --- | --- | --- | --- | --- | --- | --- | --- | --- | --- | --- | --- | --- |
| M | 60 | 61 | 62 | 64 | 65 | 66 | 68 | 69 | 70 | 71 | M | 72 | 73 | 74 | 75 | 76 | 77 | 78 | 79 | 80 | 82 | N | P Ed | P Eh |
| --- | --- | --- | --- | --- | --- | --- | --- | --- | --- | --- | --- | --- | --- | --- | --- | --- | --- | --- | --- | --- | --- | --- | --- | --- |
| M | 83 | 84 | 85 | 86 | 87 | 88 | 89 | 90 | 92 | 93 | M | 94 | 95 | 96 | 97 | 99 | 101 | 102 | 103 | 104 | 105 | N | P Ed | P Eh |
| --- | --- | --- | --- | --- | --- | --- | --- | --- | --- | --- | --- | --- | --- | --- | --- | --- | --- | --- | --- | --- | --- | --- | --- | --- |

## Slide 4
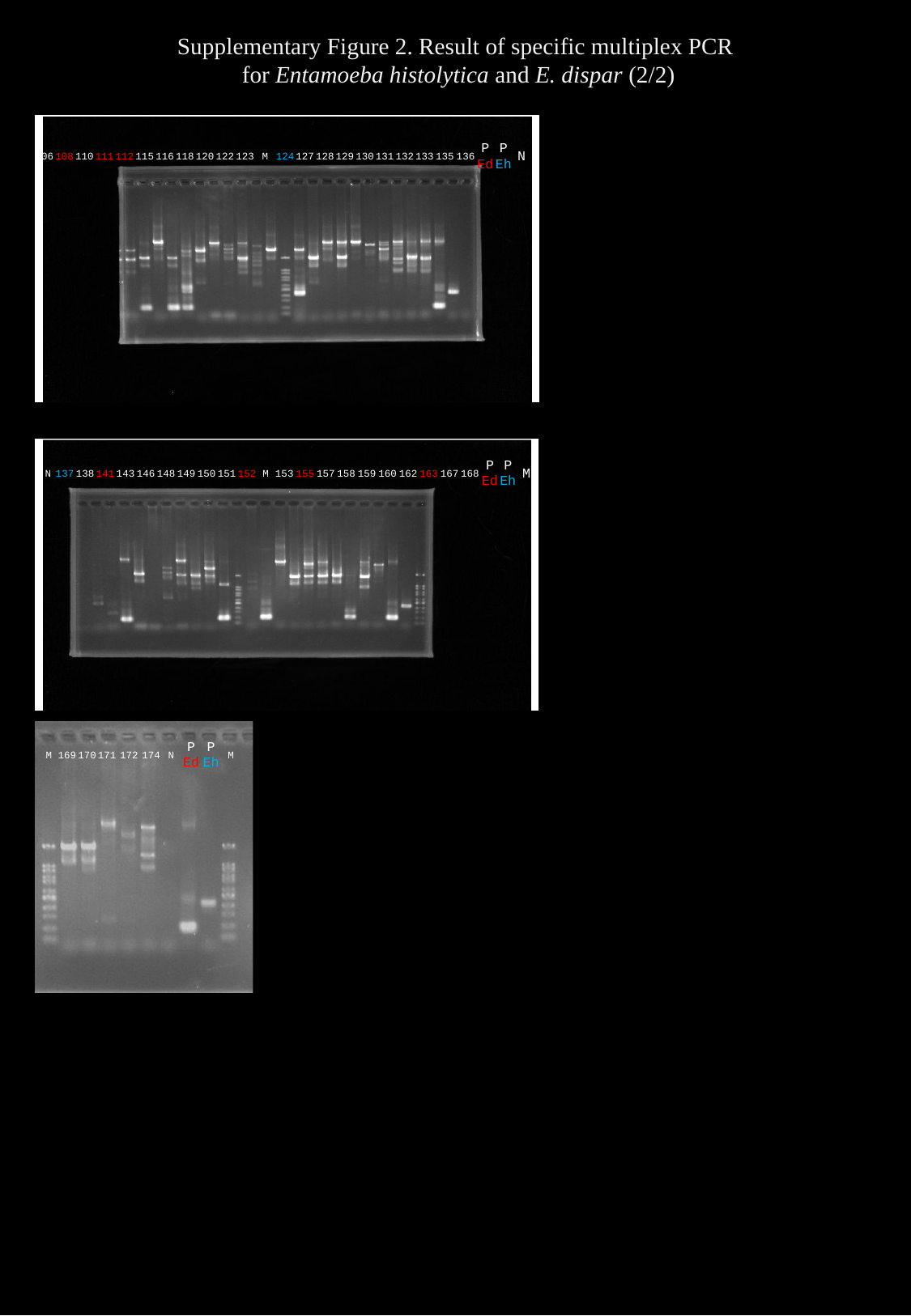

Supplementary Figure 2. Result of specific multiplex PCR
for Entamoeba histolytica and E. dispar (2/2)
| 106 | 108 | 110 | 111 | 112 | 115 | 116 | 118 | 120 | 122 | 123 | M | 124 | 127 | 128 | 129 | 130 | 131 | 132 | 133 | 135 | 136 | P Ed | P Eh | N |
| --- | --- | --- | --- | --- | --- | --- | --- | --- | --- | --- | --- | --- | --- | --- | --- | --- | --- | --- | --- | --- | --- | --- | --- | --- |
| N | 137 | 138 | 141 | 143 | 146 | 148 | 149 | 150 | 151 | 152 | M | 153 | 155 | 157 | 158 | 159 | 160 | 162 | 163 | 167 | 168 | P Ed | P Eh | M |
| --- | --- | --- | --- | --- | --- | --- | --- | --- | --- | --- | --- | --- | --- | --- | --- | --- | --- | --- | --- | --- | --- | --- | --- | --- |
| M | 169 | 170 | 171 | 172 | 174 | N | P Ed | P Eh | M |
| --- | --- | --- | --- | --- | --- | --- | --- | --- | --- |

## Slide 5
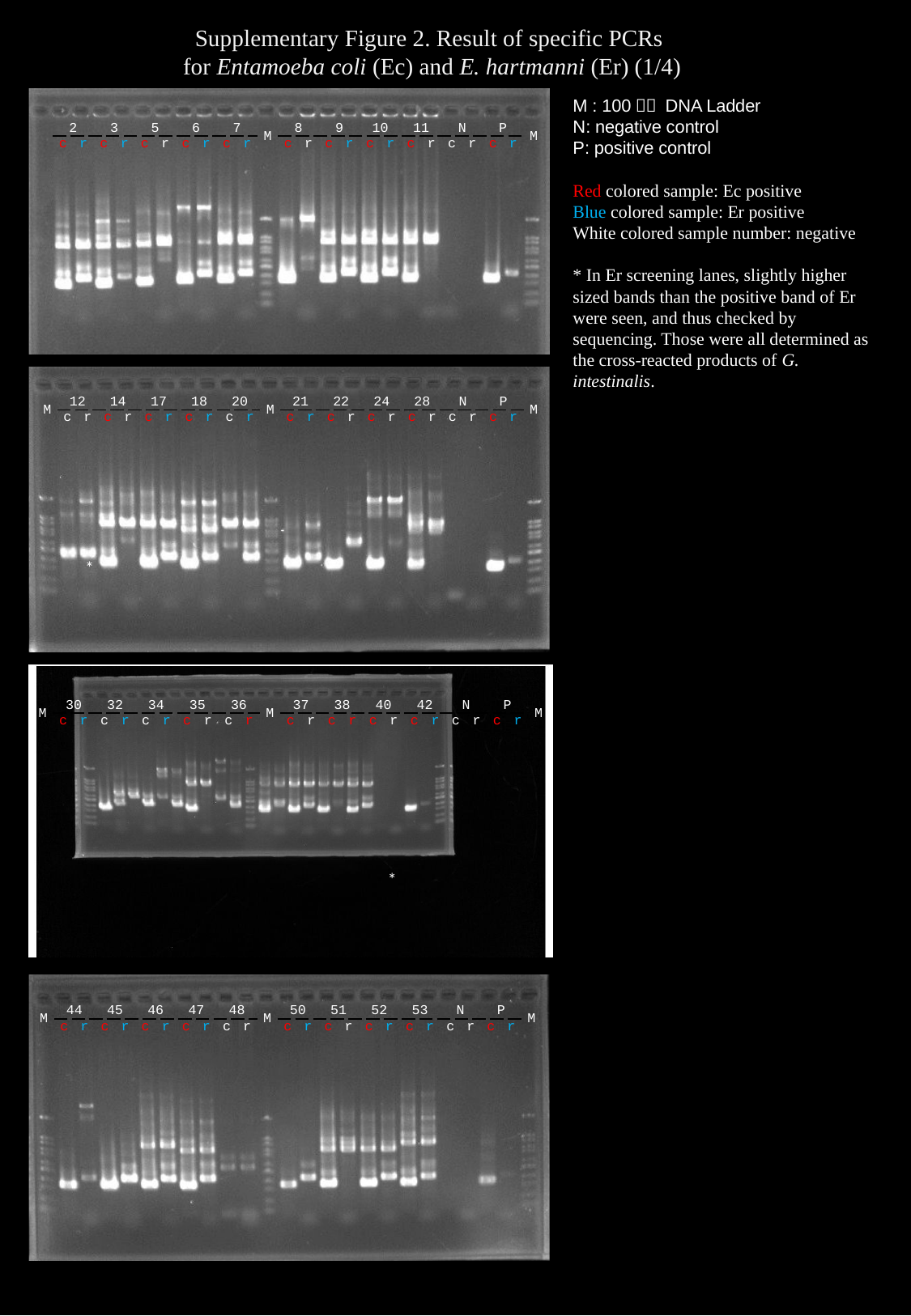

# Supplementary Figure 2. Result of specific PCRs for Entamoeba coli (Ec) and E. hartmanni (Er) (1/4)
M : 100ｂｐ DNA Ladder
N: negative control
P: positive control
Red colored sample: Ec positive
Blue colored sample: Er positive
White colored sample number: negative
* In Er screening lanes, slightly higher sized bands than the positive band of Er were seen, and thus checked by sequencing. Those were all determined as the cross-reacted products of G. intestinalis.
| | 2 | | 3 | | 5 | | 6 | | 7 | | M | 8 | | 9 | | 10 | | 11 | | N | | P | | M |
| --- | --- | --- | --- | --- | --- | --- | --- | --- | --- | --- | --- | --- | --- | --- | --- | --- | --- | --- | --- | --- | --- | --- | --- | --- |
| | c | r | c | r | c | r | c | r | c | r | | c | r | c | r | c | r | c | r | c | r | c | r | |
| M | 12 | | 14 | | 17 | | 18 | | 20 | | M | 21 | | 22 | | 24 | | 28 | | N | | P | | M |
| --- | --- | --- | --- | --- | --- | --- | --- | --- | --- | --- | --- | --- | --- | --- | --- | --- | --- | --- | --- | --- | --- | --- | --- | --- |
| | c | r | c | r | c | r | c | r | c | r | | c | r | c | r | c | r | c | r | c | r | c | r | |
*
| M | 30 | | 32 | | 34 | | 35 | | 36 | | M | 37 | | 38 | | 40 | | 42 | | N | | P | | M |
| --- | --- | --- | --- | --- | --- | --- | --- | --- | --- | --- | --- | --- | --- | --- | --- | --- | --- | --- | --- | --- | --- | --- | --- | --- |
| | c | r | c | r | c | r | c | r | c | r | | c | r | c | r | c | r | c | r | c | r | c | r | |
*
| M | 44 | | 45 | | 46 | | 47 | | 48 | | M | 50 | | 51 | | 52 | | 53 | | N | | P | | M |
| --- | --- | --- | --- | --- | --- | --- | --- | --- | --- | --- | --- | --- | --- | --- | --- | --- | --- | --- | --- | --- | --- | --- | --- | --- |
| | c | r | c | r | c | r | c | r | c | r | | c | r | c | r | c | r | c | r | c | r | c | r | |

## Slide 6
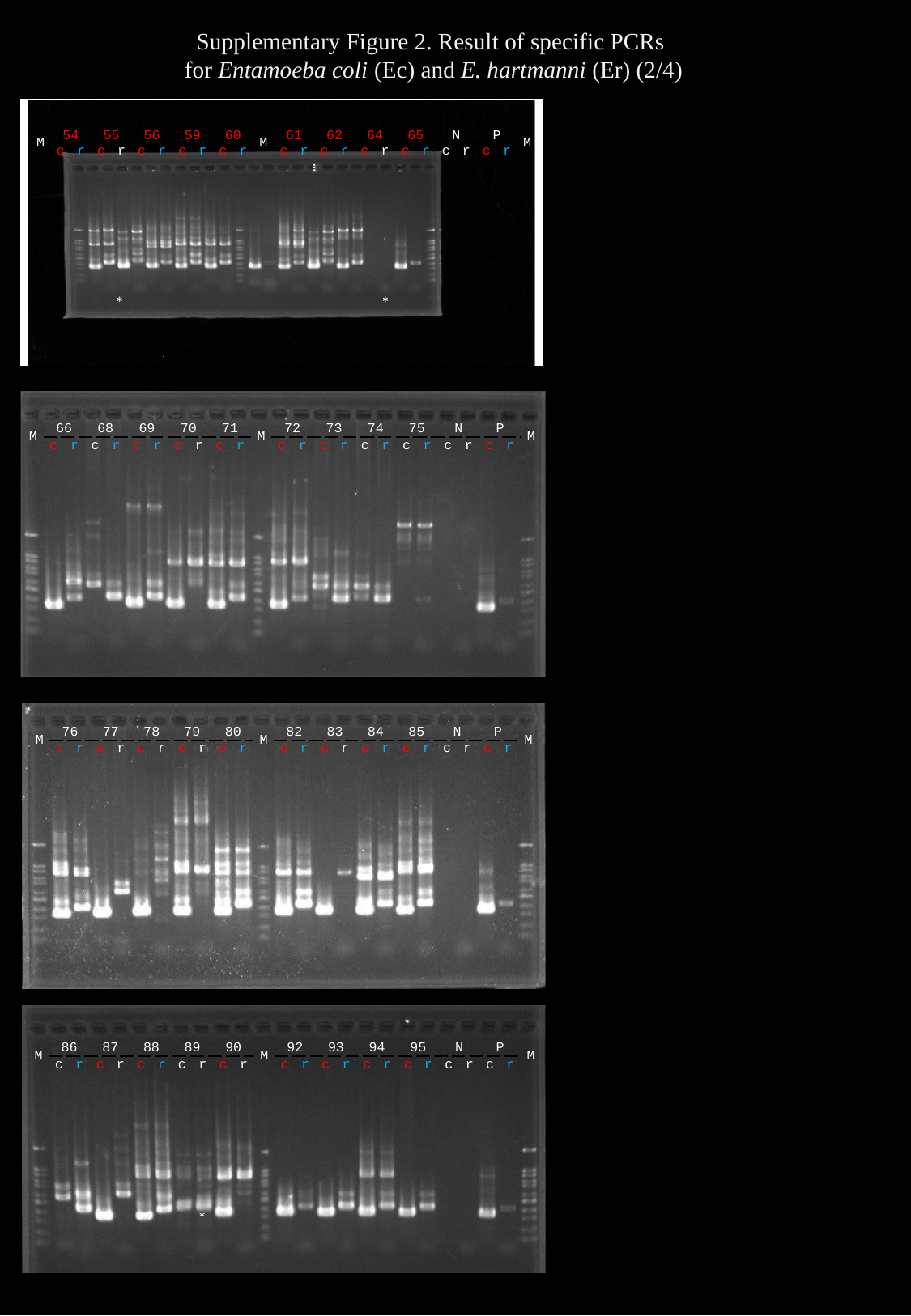

# Supplementary Figure 2. Result of specific PCRs for Entamoeba coli (Ec) and E. hartmanni (Er) (2/4)
| M | 54 | | 55 | | 56 | | 59 | | 60 | | M | 61 | | 62 | | 64 | | 65 | | N | | P | | M |
| --- | --- | --- | --- | --- | --- | --- | --- | --- | --- | --- | --- | --- | --- | --- | --- | --- | --- | --- | --- | --- | --- | --- | --- | --- |
| | c | r | c | r | c | r | c | r | c | r | | c | r | c | r | c | r | c | r | c | r | c | r | |
*
*
| M | 66 | | 68 | | 69 | | 70 | | 71 | | M | 72 | | 73 | | 74 | | 75 | | N | | P | | M |
| --- | --- | --- | --- | --- | --- | --- | --- | --- | --- | --- | --- | --- | --- | --- | --- | --- | --- | --- | --- | --- | --- | --- | --- | --- |
| | c | r | c | r | c | r | c | r | c | r | | c | r | c | r | c | r | c | r | c | r | c | r | |
| M | 76 | | 77 | | 78 | | 79 | | 80 | | M | 82 | | 83 | | 84 | | 85 | | N | | P | | M |
| --- | --- | --- | --- | --- | --- | --- | --- | --- | --- | --- | --- | --- | --- | --- | --- | --- | --- | --- | --- | --- | --- | --- | --- | --- |
| | c | r | c | r | c | r | c | r | c | r | | c | r | c | r | c | r | c | r | c | r | c | r | |
| M | 86 | | 87 | | 88 | | 89 | | 90 | | M | 92 | | 93 | | 94 | | 95 | | N | | P | | M |
| --- | --- | --- | --- | --- | --- | --- | --- | --- | --- | --- | --- | --- | --- | --- | --- | --- | --- | --- | --- | --- | --- | --- | --- | --- |
| | c | r | c | r | c | r | c | r | c | r | | c | r | c | r | c | r | c | r | c | r | c | r | |
*

## Slide 7
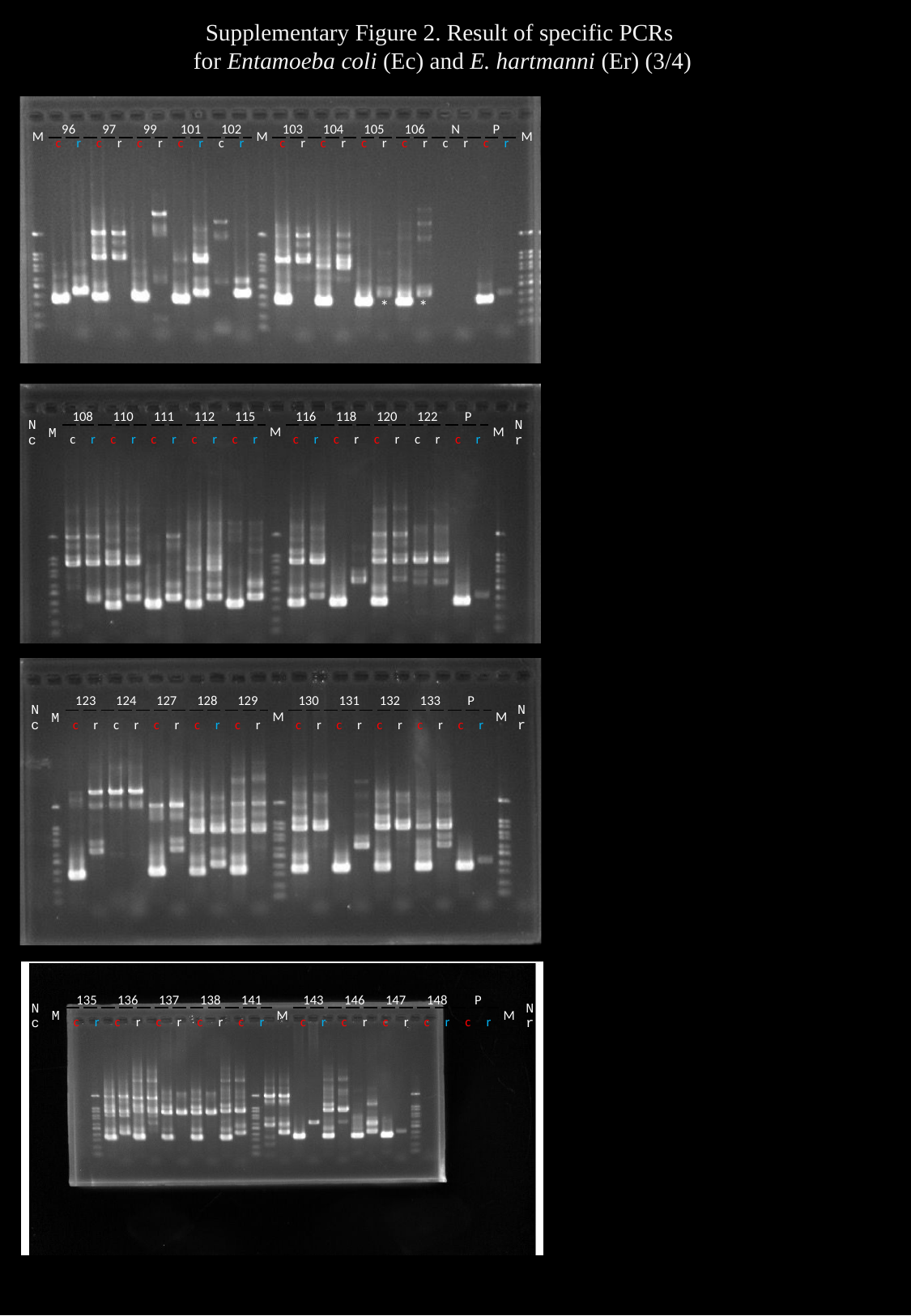

# Supplementary Figure 2. Result of specific PCRs for Entamoeba coli (Ec) and E. hartmanni (Er) (3/4)
| M | 96 | | 97 | | 99 | | 101 | | 102 | | M | 103 | | 104 | | 105 | | 106 | | N | | P | | M |
| --- | --- | --- | --- | --- | --- | --- | --- | --- | --- | --- | --- | --- | --- | --- | --- | --- | --- | --- | --- | --- | --- | --- | --- | --- |
| | c | r | c | r | c | r | c | r | c | r | | c | r | c | r | c | r | c | r | c | r | c | r | |
*
*
| N c | M | 108 | | 110 | | 111 | | 112 | | 115 | | M | 116 | | 118 | | 120 | | 122 | | P | | M | N r |
| --- | --- | --- | --- | --- | --- | --- | --- | --- | --- | --- | --- | --- | --- | --- | --- | --- | --- | --- | --- | --- | --- | --- | --- | --- |
| | | c | r | c | r | c | r | c | r | c | r | | c | r | c | r | c | r | c | r | c | r | | |
| N c | M | 123 | | 124 | | 127 | | 128 | | 129 | | M | 130 | | 131 | | 132 | | 133 | | P | | M | N r |
| --- | --- | --- | --- | --- | --- | --- | --- | --- | --- | --- | --- | --- | --- | --- | --- | --- | --- | --- | --- | --- | --- | --- | --- | --- |
| | | c | r | c | r | c | r | c | r | c | r | | c | r | c | r | c | r | c | r | c | r | | |
| N c | M | 135 | | 136 | | 137 | | 138 | | 141 | | M | 143 | | 146 | | 147 | | 148 | | P | | M | N r |
| --- | --- | --- | --- | --- | --- | --- | --- | --- | --- | --- | --- | --- | --- | --- | --- | --- | --- | --- | --- | --- | --- | --- | --- | --- |
| | | c | r | c | r | c | r | c | r | c | r | | c | r | c | r | c | r | c | r | c | r | | |

## Slide 8
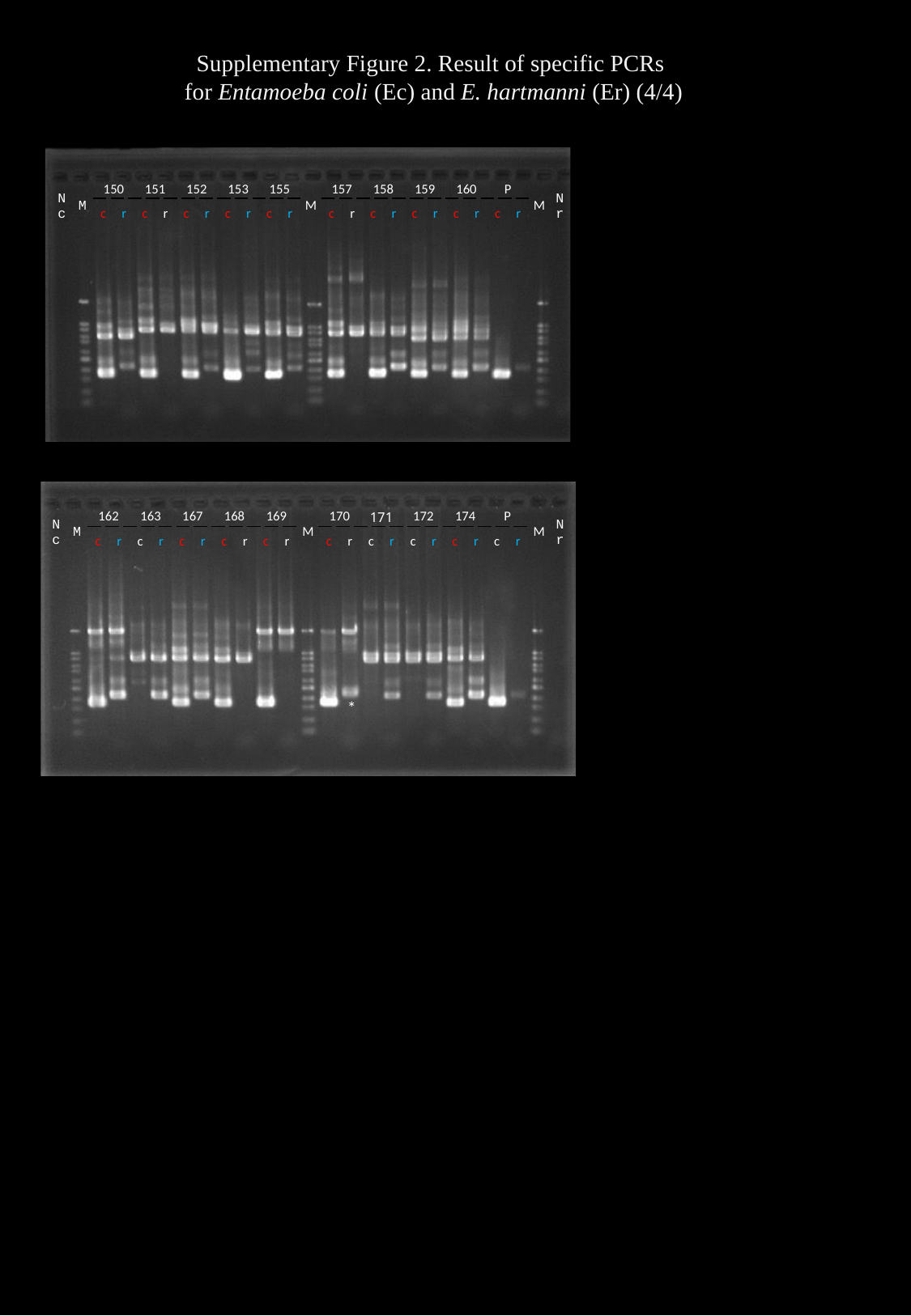

# Supplementary Figure 2. Result of specific PCRs for Entamoeba coli (Ec) and E. hartmanni (Er) (4/4)
| N c | M | 150 | | 151 | | 152 | | 153 | | 155 | | M | 157 | | 158 | | 159 | | 160 | | P | | M | N r |
| --- | --- | --- | --- | --- | --- | --- | --- | --- | --- | --- | --- | --- | --- | --- | --- | --- | --- | --- | --- | --- | --- | --- | --- | --- |
| | | c | r | c | r | c | r | c | r | c | r | | c | r | c | r | c | r | c | r | c | r | | |
| N c | M | 162 | | 163 | | 167 | | 168 | | 169 | | M | 170 | | 171 | | 172 | | 174 | | P | | M | N r |
| --- | --- | --- | --- | --- | --- | --- | --- | --- | --- | --- | --- | --- | --- | --- | --- | --- | --- | --- | --- | --- | --- | --- | --- | --- |
| | | c | r | c | r | c | r | c | r | c | r | | c | r | c | r | c | r | c | r | c | r | | |
*

## Slide 9
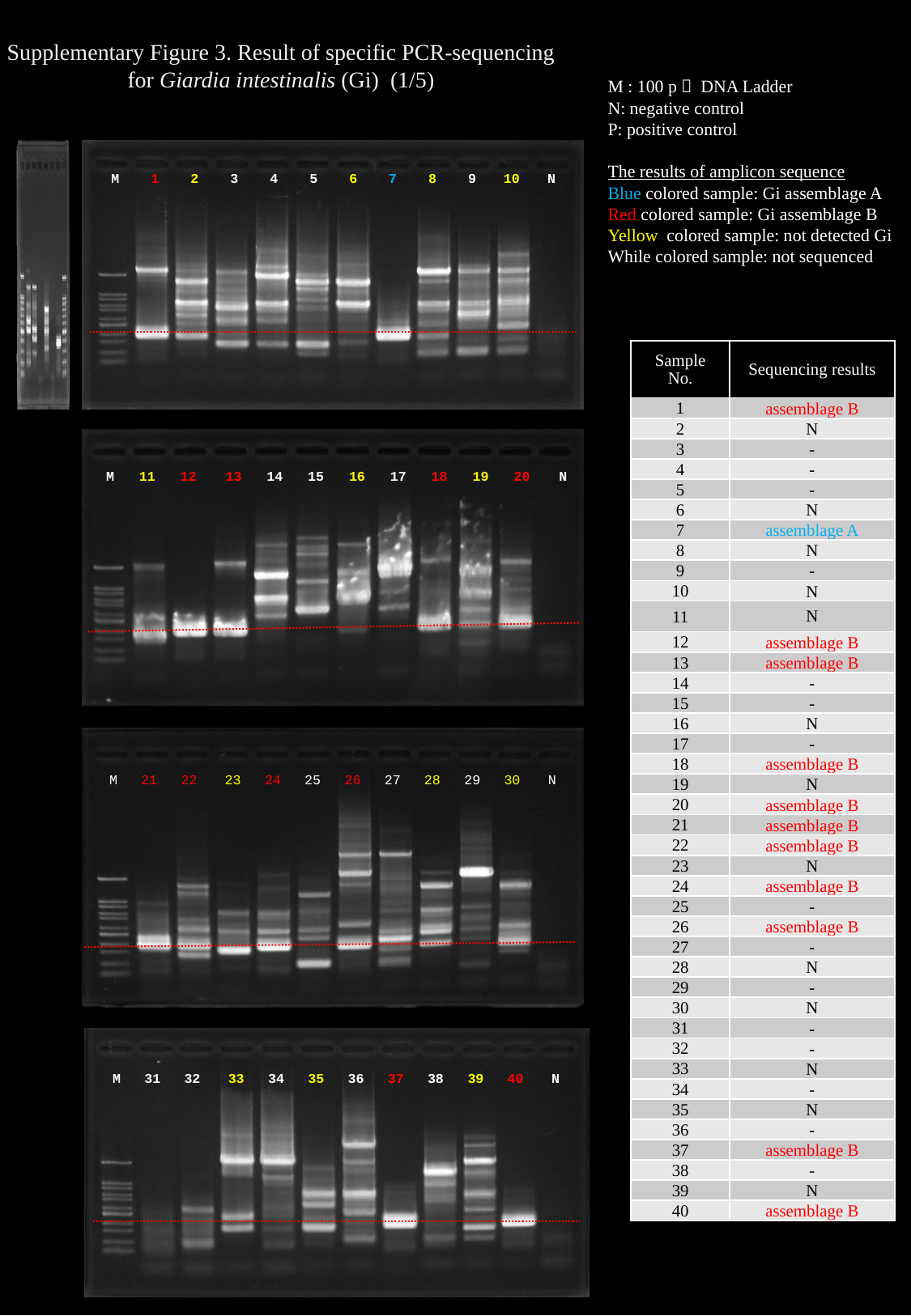

Supplementary Figure 3. Result of specific PCR-sequencing
for Giardia intestinalis (Gi) (1/5)
M : 100 pｂ DNA Ladder
N: negative control
P: positive control
The results of amplicon sequence
Blue colored sample: Gi assemblage A
Red colored sample: Gi assemblage B
Yellow colored sample: not detected Gi
While colored sample: not sequenced
| M | 1 | 2 | 3 | 4 | 5 | 6 | 7 | 8 | 9 | 10 | N |
| --- | --- | --- | --- | --- | --- | --- | --- | --- | --- | --- | --- |
| Sample No. | Sequencing results |
| --- | --- |
| 1 | assemblage B |
| 2 | N |
| 3 | - |
| 4 | - |
| 5 | - |
| 6 | N |
| 7 | assemblage A |
| 8 | N |
| 9 | - |
| 10 | N |
| 11 | N |
| 12 | assemblage B |
| 13 | assemblage B |
| 14 | - |
| 15 | - |
| 16 | N |
| 17 | - |
| 18 | assemblage B |
| 19 | N |
| 20 | assemblage B |
| 21 | assemblage B |
| 22 | assemblage B |
| 23 | N |
| 24 | assemblage B |
| 25 | - |
| 26 | assemblage B |
| 27 | - |
| 28 | N |
| 29 | - |
| 30 | N |
| 31 | - |
| 32 | - |
| 33 | N |
| 34 | - |
| 35 | N |
| 36 | - |
| 37 | assemblage B |
| 38 | - |
| 39 | N |
| 40 | assemblage B |
| M | 11 | 12 | 13 | 14 | 15 | 16 | 17 | 18 | 19 | 20 | N |
| --- | --- | --- | --- | --- | --- | --- | --- | --- | --- | --- | --- |
| M | 21 | 22 | 23 | 24 | 25 | 26 | 27 | 28 | 29 | 30 | N |
| --- | --- | --- | --- | --- | --- | --- | --- | --- | --- | --- | --- |
| M | 31 | 32 | 33 | 34 | 35 | 36 | 37 | 38 | 39 | 40 | N |
| --- | --- | --- | --- | --- | --- | --- | --- | --- | --- | --- | --- |

## Slide 10
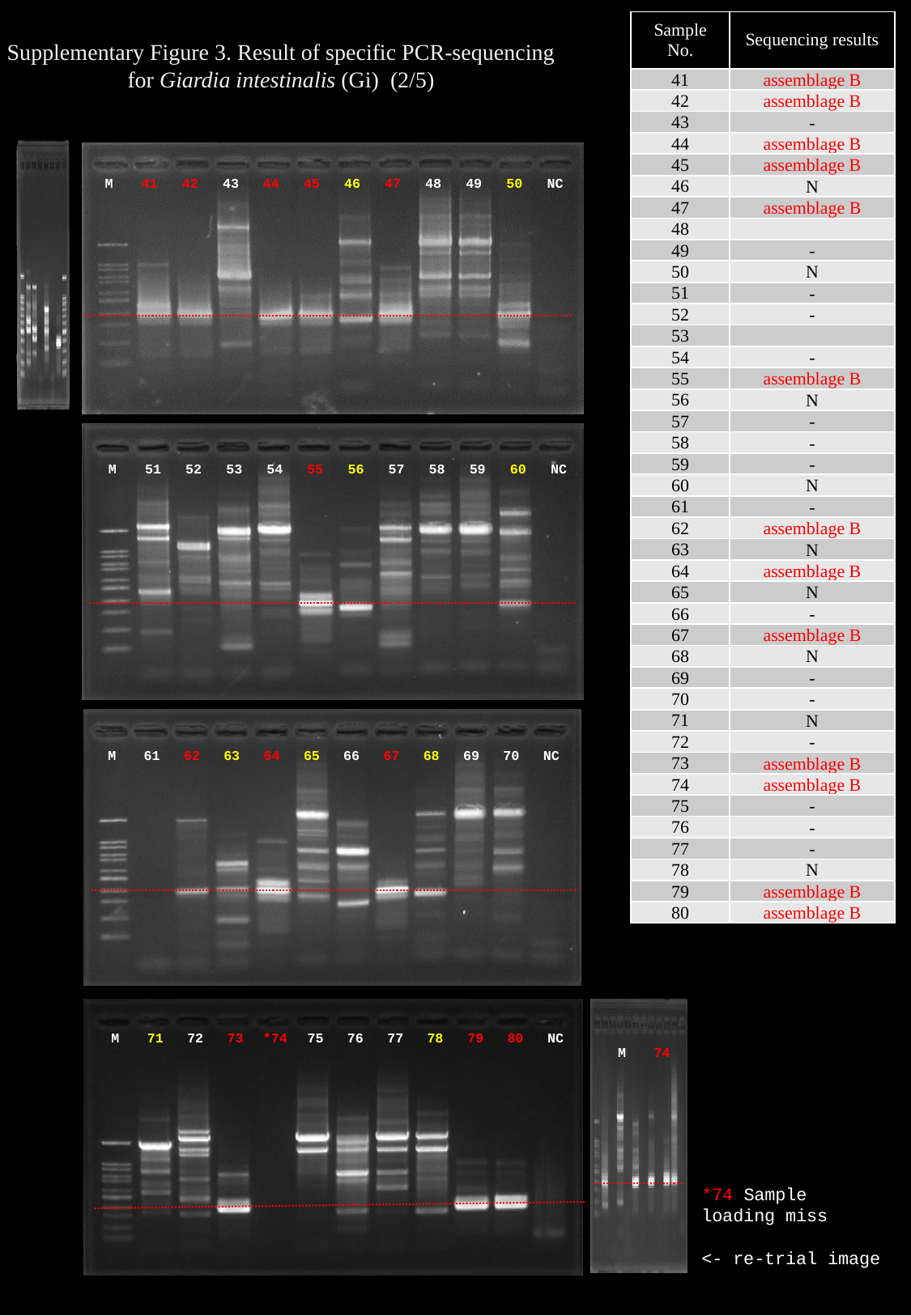

| Sample No. | Sequencing results |
| --- | --- |
| 41 | assemblage B |
| 42 | assemblage B |
| 43 | - |
| 44 | assemblage B |
| 45 | assemblage B |
| 46 | N |
| 47 | assemblage B |
| 48 | |
| 49 | - |
| 50 | N |
| 51 | - |
| 52 | - |
| 53 | |
| 54 | - |
| 55 | assemblage B |
| 56 | N |
| 57 | - |
| 58 | - |
| 59 | - |
| 60 | N |
| 61 | - |
| 62 | assemblage B |
| 63 | N |
| 64 | assemblage B |
| 65 | N |
| 66 | - |
| 67 | assemblage B |
| 68 | N |
| 69 | - |
| 70 | - |
| 71 | N |
| 72 | - |
| 73 | assemblage B |
| 74 | assemblage B |
| 75 | - |
| 76 | - |
| 77 | - |
| 78 | N |
| 79 | assemblage B |
| 80 | assemblage B |
Supplementary Figure 3. Result of specific PCR-sequencing
for Giardia intestinalis (Gi) (2/5)
| M | 41 | 42 | 43 | 44 | 45 | 46 | 47 | 48 | 49 | 50 | NC |
| --- | --- | --- | --- | --- | --- | --- | --- | --- | --- | --- | --- |
| M | 51 | 52 | 53 | 54 | 55 | 56 | 57 | 58 | 59 | 60 | NC |
| --- | --- | --- | --- | --- | --- | --- | --- | --- | --- | --- | --- |
| M | 61 | 62 | 63 | 64 | 65 | 66 | 67 | 68 | 69 | 70 | NC |
| --- | --- | --- | --- | --- | --- | --- | --- | --- | --- | --- | --- |
| M | 71 | 72 | 73 | \*74 | 75 | 76 | 77 | 78 | 79 | 80 | NC |
| --- | --- | --- | --- | --- | --- | --- | --- | --- | --- | --- | --- |
| M | 74 |
| --- | --- |
*74 Sample loading miss
<- re-trial image

## Slide 11
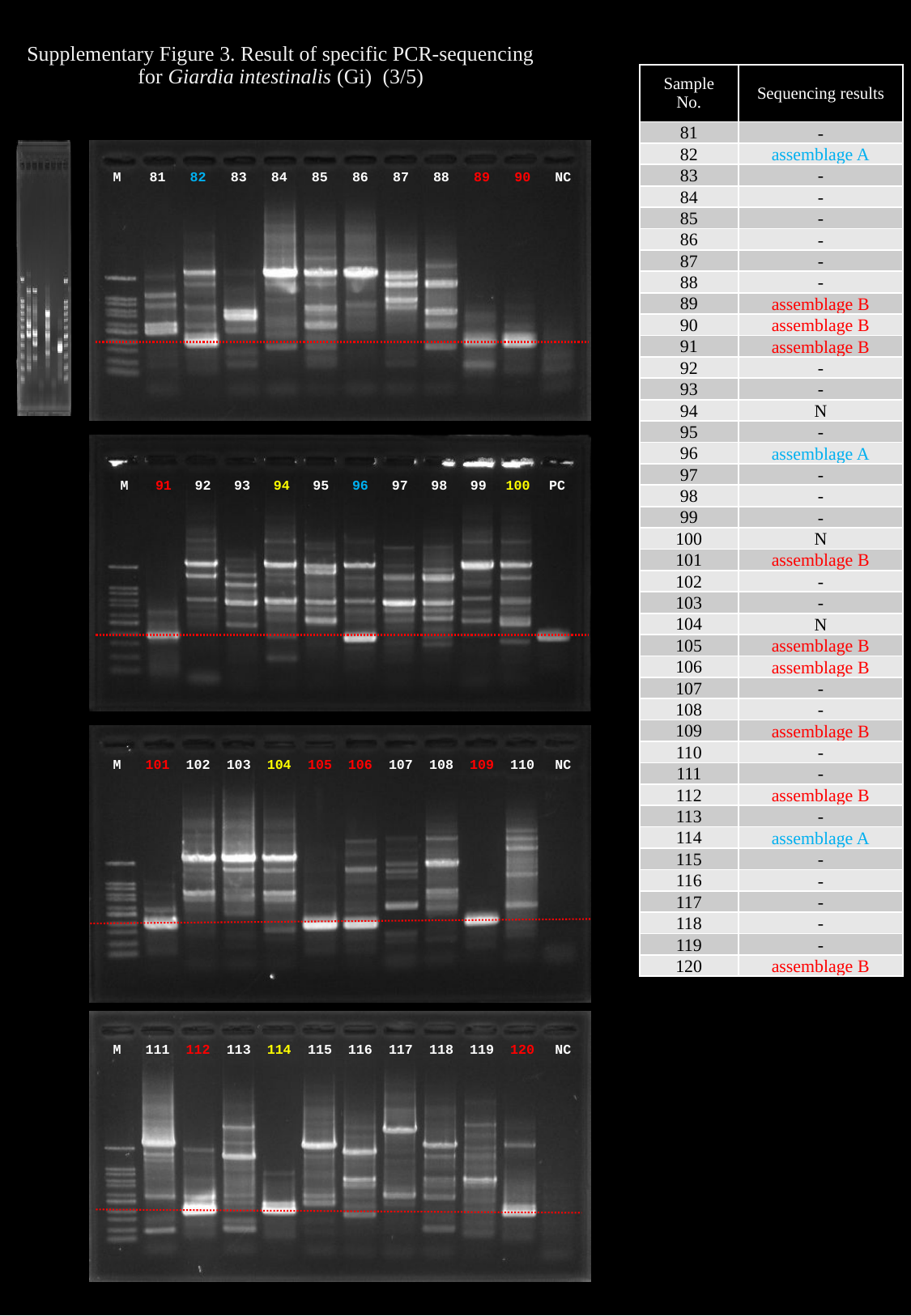

Supplementary Figure 3. Result of specific PCR-sequencing
for Giardia intestinalis (Gi) (3/5)
| Sample No. | Sequencing results |
| --- | --- |
| 81 | - |
| 82 | assemblage A |
| 83 | - |
| 84 | - |
| 85 | - |
| 86 | - |
| 87 | - |
| 88 | - |
| 89 | assemblage B |
| 90 | assemblage B |
| 91 | assemblage B |
| 92 | - |
| 93 | - |
| 94 | N |
| 95 | - |
| 96 | assemblage A |
| 97 | - |
| 98 | - |
| 99 | - |
| 100 | N |
| 101 | assemblage B |
| 102 | - |
| 103 | - |
| 104 | N |
| 105 | assemblage B |
| 106 | assemblage B |
| 107 | - |
| 108 | - |
| 109 | assemblage B |
| 110 | - |
| 111 | - |
| 112 | assemblage B |
| 113 | - |
| 114 | assemblage A |
| 115 | - |
| 116 | - |
| 117 | - |
| 118 | - |
| 119 | - |
| 120 | assemblage B |
| M | 81 | 82 | 83 | 84 | 85 | 86 | 87 | 88 | 89 | 90 | NC |
| --- | --- | --- | --- | --- | --- | --- | --- | --- | --- | --- | --- |
| M | 91 | 92 | 93 | 94 | 95 | 96 | 97 | 98 | 99 | 100 | PC |
| --- | --- | --- | --- | --- | --- | --- | --- | --- | --- | --- | --- |
| M | 101 | 102 | 103 | 104 | 105 | 106 | 107 | 108 | 109 | 110 | NC |
| --- | --- | --- | --- | --- | --- | --- | --- | --- | --- | --- | --- |
| M | 111 | 112 | 113 | 114 | 115 | 116 | 117 | 118 | 119 | 120 | NC |
| --- | --- | --- | --- | --- | --- | --- | --- | --- | --- | --- | --- |

## Slide 12
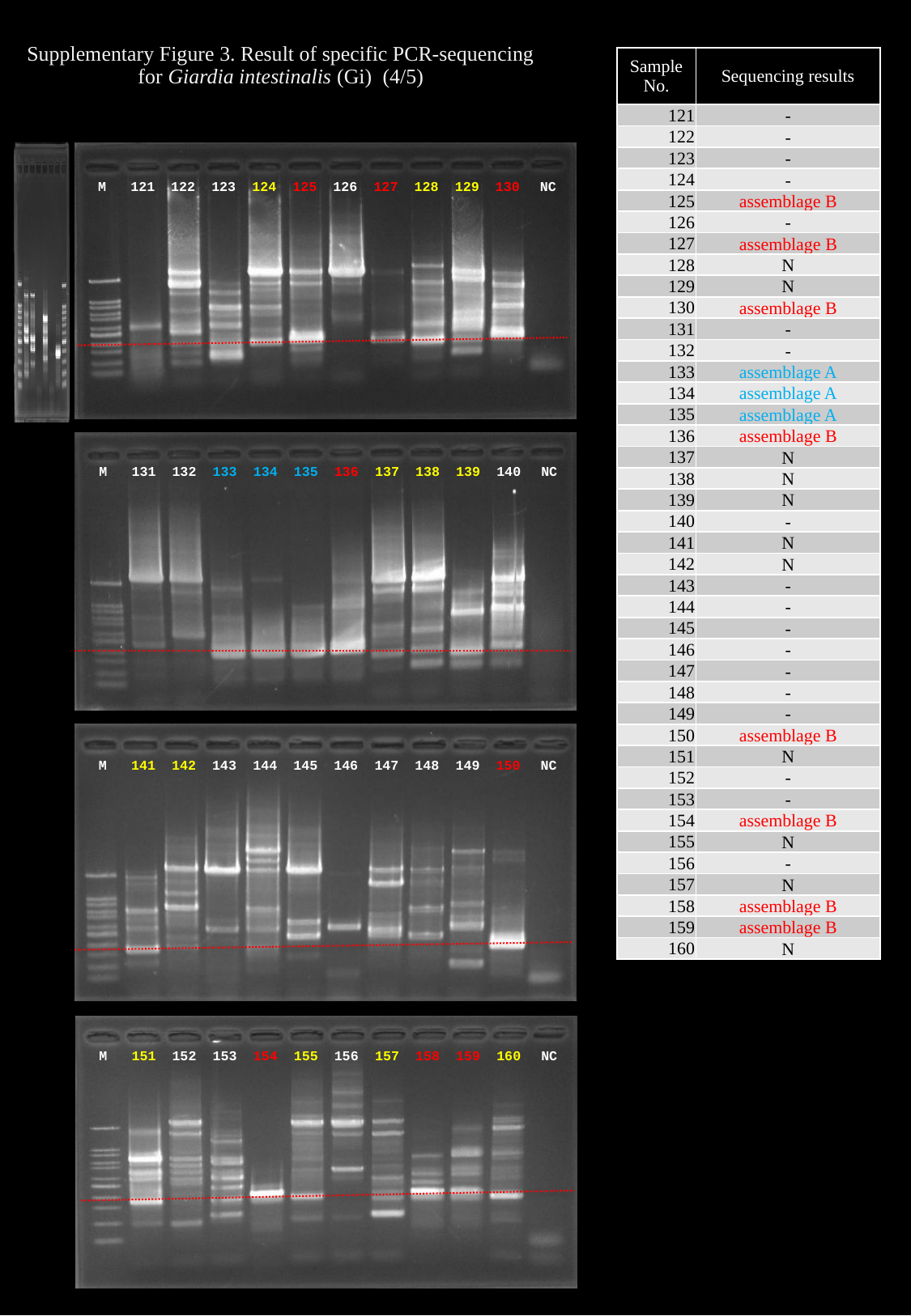

Supplementary Figure 3. Result of specific PCR-sequencing
for Giardia intestinalis (Gi) (4/5)
| Sample No. | Sequencing results |
| --- | --- |
| 121 | - |
| 122 | - |
| 123 | - |
| 124 | - |
| 125 | assemblage B |
| 126 | - |
| 127 | assemblage B |
| 128 | N |
| 129 | N |
| 130 | assemblage B |
| 131 | - |
| 132 | - |
| 133 | assemblage A |
| 134 | assemblage A |
| 135 | assemblage A |
| 136 | assemblage B |
| 137 | N |
| 138 | N |
| 139 | N |
| 140 | - |
| 141 | N |
| 142 | N |
| 143 | - |
| 144 | - |
| 145 | - |
| 146 | - |
| 147 | - |
| 148 | - |
| 149 | - |
| 150 | assemblage B |
| 151 | N |
| 152 | - |
| 153 | - |
| 154 | assemblage B |
| 155 | N |
| 156 | - |
| 157 | N |
| 158 | assemblage B |
| 159 | assemblage B |
| 160 | N |
| M | 121 | 122 | 123 | 124 | 125 | 126 | 127 | 128 | 129 | 130 | NC |
| --- | --- | --- | --- | --- | --- | --- | --- | --- | --- | --- | --- |
| M | 131 | 132 | 133 | 134 | 135 | 136 | 137 | 138 | 139 | 140 | NC |
| --- | --- | --- | --- | --- | --- | --- | --- | --- | --- | --- | --- |
| M | 141 | 142 | 143 | 144 | 145 | 146 | 147 | 148 | 149 | 150 | NC |
| --- | --- | --- | --- | --- | --- | --- | --- | --- | --- | --- | --- |
| M | 151 | 152 | 153 | 154 | 155 | 156 | 157 | 158 | 159 | 160 | NC |
| --- | --- | --- | --- | --- | --- | --- | --- | --- | --- | --- | --- |

## Slide 13
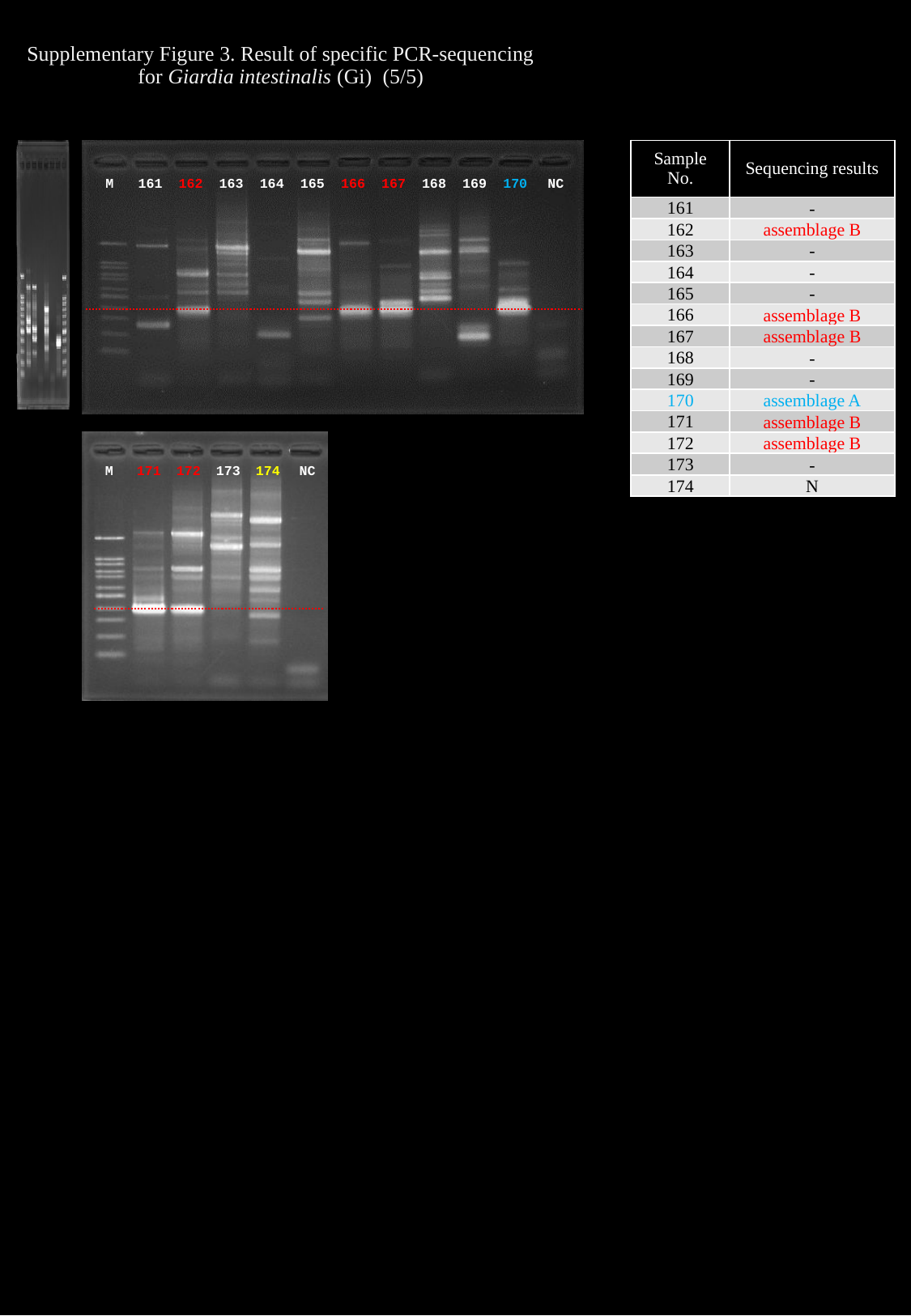

Supplementary Figure 3. Result of specific PCR-sequencing
for Giardia intestinalis (Gi) (5/5)
| Sample No. | Sequencing results |
| --- | --- |
| 161 | - |
| 162 | assemblage B |
| 163 | - |
| 164 | - |
| 165 | - |
| 166 | assemblage B |
| 167 | assemblage B |
| 168 | - |
| 169 | - |
| 170 | assemblage A |
| 171 | assemblage B |
| 172 | assemblage B |
| 173 | - |
| 174 | N |
| M | 161 | 162 | 163 | 164 | 165 | 166 | 167 | 168 | 169 | 170 | NC |
| --- | --- | --- | --- | --- | --- | --- | --- | --- | --- | --- | --- |
| M | 171 | 172 | 173 | 174 | NC |
| --- | --- | --- | --- | --- | --- |
